# Identification of the group IIa WRKY subfamily and the functional analysis of *GhWRKY17* in upland cotton (*Gossypium hirsutum* L.)

**DOI:** 10.1371/journal.pone.0191681

**Published:** 2018-01-25

**Authors:** Lijiao Gu, Libei Li, Hengling Wei, Hantao Wang, Junji Su, Yaning Guo, Shuxun Yu

**Affiliations:** 1 State Key Laboratory of Cotton Biology, Institute of Cotton Research of CAAS, Anyang, Henan, China; 2 College of Agronomy, Northwest A&F University, Yangling, Shanxi, China; Wuhan University, CHINA

## Abstract

WRKY transcription factors play important roles in plant defense, stress response, leaf senescence, and plant growth and development. Previous studies have revealed the important roles of the group IIa *GhWRKY* genes in cotton. To comprehensively analyze the group IIa *GhWRKY* genes in upland cotton, we identified 15 candidate group IIa *GhWRKY* genes in the *Gossypium hirsutum* genome. The phylogenetic tree, intron-exon structure, motif prediction and Ka/Ks analyses indicated that most group IIa *GhWRKY* genes shared high similarity and conservation and underwent purifying selection during evolution. In addition, we detected the expression patterns of several group IIa *GhWRKY* genes in individual tissues as well as during leaf senescence using public RNA sequencing data and real-time quantitative PCR. To better understand the functions of group IIa *GhWRKYs* in cotton, *GhWRKY17* (KF669857) was isolated from upland cotton, and its sequence alignment, promoter *cis*-acting elements and subcellular localization were characterized. Moreover, the over-expression of *GhWRKY17* in *Arabidopsis* up-regulated the senescence-associated genes *AtWRKY53*, *AtSAG12* and *AtSAG13*, enhancing the plant’s susceptibility to leaf senescence. These findings lay the foundation for further analysis and study of the functions of WRKY genes in cotton.

## Introduction

Throughout their life cycle, plants exhibit a set of complex adjustment mechanisms to perceive and respond to various physiological and developmental signals. Among these diverse adjustment mechanisms, transcriptional regulation mechanisms, which are mainly executed by transcription factors (TFs), play important roles [1]. For example, WRKY proteins constitute one of the largest TF families in plants [2]. Since the first WRKY gene, *SPF1*, was reported in sweet potato [3], increasing numbers of WRKY genes have been reported in various species, including *Arabidopsis thaliana*, *Gossypium hirsutum*, *Oryza sativa*, *Ricinus communis*, *Manihot esculenta* and *Cucumis sativus* [4–9].

WRKY TFs were named for their conserved WRKY domain, which consists of approximately 60 amino acids containing a conserved WRKYGQK core sequence and a zinc finger-like motif [2]. The WRKY TF family is divided into three main groups according to the number of WRKY domains and the pattern of zinc finger motifs: group I contains two WRKY domains each with a C2H2 zinc finger motif, whereas group II and group III each contain a single WRKY domain with either a C2H2 zinc finger motif or a C2HC zinc finger motif, respectively. However, group II can be further divided into five subgroups (IIa, IIb, IIc, IId, and IIe) according to the amino acid motifs outside the WRKY domain [2,10–13]. The group IIa WRKY domain possesses a conserved VQR intron in the zinc finger motif nearer to the C-terminus [14].

Previous studies have reported 3 group IIa WRKY genes in *Arabidopsis thaliana*, 4 in *Oryza sativa*, 6 in *Gossypium hirsutum*, 5 in *Populus trichocarpa* and 4 in *Cucumis sativus* [9,15,5]. Group IIa WRKY genes appear to include a small number of members, but they participate widely in the regulation of diverse physiological processes, such as defense, trichome development and leaf senescence [16,17,13,18]. In *Arabidopsis*, the genes *AtWRKY18*, *AtWRKY40* and *AtWRKY60* represent the group IIa WRKY subfamily [2]. These three homologs exhibit a complex pattern of physical and functional interactions in response to the microbial pathogens *Pseudomonas syringae* and *Botrytis cinerea* [19]. In addition, the over-expression of *AtWRKY18* in *Arabidopsis* can delay leaf senescence, but *AtWRKY18* T-DNA insertion lines show an early leaf senescence phenotype. An *AtWRKY18*-*AtWRKY53*-mediated signaling pathway is involved in the senescence process [20]. However, more work has focused on the role of these three genes in the abscisic acid (ABA) signaling pathway. For example, the three WRKY genes were identified as negative regulators of ABA signaling [21,22] and can bind to W-box elements in the promoter regions of *ABI4* and *ABI5* to inhibit the expression of these two genes [23]. In rice, *OsWRKY28*, *OsWRKY62*, *OsWRKY71* and *OsWRKY76* are four members of the *OsWRKY* group IIa subfamily and are involved in modulating plant innate immunity [24]. *OsWRKY28* plays a negative regulatory role in the resistance to rice blast fungus Ina86-137, as determined by phenotypic analysis of an *oswrky28* mutant [25]. *OsWRKY71* was found to be induced by salicylic acid (SA), methyl jasmonic acid (MeJA) and pathogen infection [26]. *OsWRKY62* responds negatively to innate immunity in terms of susceptibility to pathogens [27]. Additionally, inducible alternative splicing of the genes *OsWRKY62* and *OsWRKY76* participates in pathogen defense, as found through the analysis of over-expression and loss-of-function knockout rice plants [28]. Furthermore, the group IIa WRKY genes have also been studied in other species. For example, the over-expression of *PtrWRKY40* can enhance resistance to the necrotrophic fungus *B*. *cinerea* in *Arabidopsis* and susceptibility to D*othiorella gregaria* in poplar [29], and *TaWRKY71-1* presents a hyponastic leaf phenotype by altering IAA levels in transgenic *Arabidopsis* [30].

Since the release of abundant genome sequences and publicly available transcriptome data for cotton [31–34], preliminary analyses of the group IIa WRKY subfamily genes have been performed. The three *Gossypium aridum* group IIa genes *GarWRKY28*, *GarWRKY51* and *GarWRKY52* are salt-responsive genes that display distinct expression levels in response to salt treatment [35]. Gene sequence analysis of the group IIa WRKY genes in *Gossypium raimondii* and *Gossypium arboreum* revealed a higher number of SNPs in intron regions than in exon regions. Based on protein sequence analysis, the WRKY domain regions were found to be more conserved than the regions outside the WRKY domain [36]. Cai *et al*. isolated 7 group IIa genes from *Gossypium raimondii*, and expression profiling showed that *GrWRKY24* and *GrWRKY40* are significantly induced by salt, drought and disease treatments [37]. The results of Dou *et al*. indicated that group IIa WRKY genes play important roles in leaf senescence, anther development, fiber growth, and abiotic and biotic stresses responses [5]. Moreover, several reports have addressed the functional and mechanistic details of group IIa WRKY genes in cotton. The stress–induced gene *GhWRKY40* enhances wounding tolerance and sensitivity to *Ralstonia solanacearum* infection in transgenic tobacco [38]. The transgenic *Arabidopsis* lines of *GarWRKY17* and *GarWRKY104* can enhance salt tolerance during different developmental stages [35]. Above all, most group IIa WRKYs have significant functions in the regulation of stress response and plant growth development.

Cotton is an important economic crop and textile material that plays important roles in the development of the national economy [39]. Previous findings have shown that stresses and senescence are important factors restricting cotton growth, fiber quality and yield. To date, the functional analysis of group IIa *GhWRKYs* has mainly focused on stress conditions, with limited analysis of their possible roles in leaf senescence. Here, we report an analysis of group IIa *GhWRKY* TFs in upland cotton pertaining to phylogeny, intron-exon structure, motif composition, Ka/Ks ratios, and expression patterns in different tissues and during leaf senescence. To further analyze the function of group IIa WRKY genes, *GhWRKY17* was isolated and characterized. *GhWRKY17* expression could be induced during the leaf senescence process and overexpression of *GhWRKY17* resulted in an early aging phenotype in transgenic *Arabidopsis*.

## Materials and methods

### Characterization of putative WRKY genes in cotton

The genome and protein sequences of *Gossypium hirsutum* were downloaded from the CottonGene database (http://www.cottongen.org) [40]. The Hidden Markov Model (HMM) profile of the WRKY domain (PF03106, WRKY.hmm) was obtained from the Pfam database (http://pfam.janelia.org). WRKY.hmm was then used to search the database for all candidate genes. The SMART program (http://smart.embl-heidelberg.de/) was employed to confirm the potential proteins according to the structural features of WRKY [41,42].

### Sequence analysis

For convenience, the gene names *GhWRKY1* to *GhWRKY239* were given based on their gene IDs in the genome database. To better clarify the evolutionary relationship and the classification of different clades, a phylogenetic tree was constructed with the MEGA 7 program [43] using the maximum likelihood method [44] or the neighbor-joining method [45]. MapChart was used to draft a chromosomal distribution sketch map of WRKY genes [46]. Diverse exon-intron structures were identified by inputting GFF3 format data on the group IIa WRKY genes into the Gene Structure Display Server (GSDS2.0) (http://gsds.cbi.pku.edu.cn/) [47]. The Multiple Em for Motif Elicitation (MEME) online software (http://meme-suite.org/tools/meme) was used to predict conserved motifs in group IIa WRKY proteins. To analyze the selective pressures among group IIa WRKY genes, Ka/Ks (nonsynonymous/synonymous) ratios were computed using the PAL2NAL web server (http://www.bork.embl.de/pal2nal/#RunP2N) [48].

### Expression pattern analysis

Public cotton RNA-Seq data were obtained from the high-throughput DNA and RNA Sequence Read Archive (SRA) of the National Center for Biotechnology Information (NCBI) (https://www.ncbi.nlm.nih.gov/). The accession numbers of the different tissues of *Gossypium hirsutum* L. acc. TM-1 were SRX797899-SRX797920 (SRA: PRJNA248163) [40]. The RNA-Seq data from *Gossypium hirsutum* during the process of leaf senescence were also analyzed. The accession numbers of new/old (new-1 and old-1) leaves from the three-leaf stage and of new/old (new-2 and old-2) leaves from the maturation/senescence stages were SRX1075619, SRX1075620, SRX1075623 and SRX1075624 [49].

### Plant materials and stress treatments

To evaluate the gene expression patterns in different tissues and during natural leaf senescence, the early-aging variety CCRI74 and the non-early-aging variety Liao4086 were planted in the cotton field at the Institute of Cotton Research of CAAS (Anyang, Henan, China). For tissue expression analysis, tissues including root, stem, leaf, petal, pistil, stamen, ovule and fiber were collected from CCRI74. To detect gene expression during natural leaf senescence, two methods were used to collect the samples. To examine different leaf development stages, the top fourth leaves from two varieties were sampled every 10 days from the 80^th^ day after sowing. To examine different leaf senescence areas in one leaf, five different stages of leaves were harvested from CCRI74: Stage 1, an expanded new leaf; Stage 2, a mature but non-senescent leaf; Stage 3, a leaf with 25% senescence area; Stage 4, a leaf with 50% senescence area; and Stage 5, a leaf with at least 75% senescence area.

For stress treatments, healthy and plump seeds of CCRI74 were planted in pots in the greenhouse at 28°C under a 16 h light/8 h dark cycle. When the seedlings had grown to the cotyledon stage, healthy and uniform 10-day-old seedlings were used for different treatments. In the exogenous hormone treatments, the seedlings were sprayed with 100 μM MeJA, 2 mM SA, 200 μM ABA and 0.5 mM ethylene released from ethephon (ETH). In the abiotic stress treatments, the seedlings were irrigated with 15% polyethylene glycol 6000 (PEG6000) and 200 mM sodium chloride (NaCl). The cotyledons were sampled at 0 h, 2 h, 4 h, 8 h, and 12 h. The samples were frozen in liquid nitrogen and used in the subsequent experiments. The experiments were repeated at least three times.

### Quantitative real-time PCR (qRT-PCR)

The total RNA was isolated using an RNAprep PurePlant Kit (Polysaccharides & Polyphenolics-rich) (Tiangen, China). The cDNA was synthesized using a PrimeScript™ RT Reagent Kit with gDNA Eraser (Perfect Real Time) (TaKaRa, Japan). qRT-PCR was performed to identify transcript levels using a 7500 Real-Time PCR System (Applied Biosystems, Foster City, CA, USA) and UltraSYBR Mixture (With ROXI) (CWBIO, China) in a 20 μl volume: 2×UltraSYBR Mixture (With ROXI) 10 μl, forward primer 0.5 μl, reverse primer 0.5 μl, template cDNA 1 μl, and RNase-free water 9 μl. The PCR procedure was as follows: a pre-denaturation step at 95°C for 10 min; 40 cycles of 95°C for 10 s, 60°C for 30 s and 72°C for 32 s; and a melting curve of 95°C for 15 s, 60°C for 1 min, 95°C for 15 s and 60°C for 15 s. The 2^−ΔΔCT^ method was applied to calculate the relative expression of genes [50]. Figures for qRT-PCR were drawn using GraphPad Prism software [51].

### Promoter cloning

The upstream 1500 bp sequence of *GhWRKY17* was obtained from the genome sequence data of upland cotton (http://www.cottongen.org) [36], and the promoter fragment was obtained from DNA by homology-based cloning [52]. Cis-acting elements in the promoter region were predicted by the online software PlantCARE (http://bioinformatics.psb.ugent.be/webtools/plantcare/html/) [53].

### Subcellular location

*GhWRKY17* protein subcellular localization was predicted by WoLF PSORT (http://www.genscript.com/wolf-psort.html). In addition, the coding region of *GhWRKY17* without the termination codon was cloned into the *pBI121-GFP* vector to construct the plasmid *pBI121-GhWRKY17-GFP* driven by the *CaMV35S* promoter. Both the recombinant plasmid *pBI121-GhWRKY17-GFP* and the empty vector *pBI121-GFP* wrapped with gold powder were transferred into onion epidermal cells cultivated on MS plates using a desk-type particle gun PDS-1000/He system (Bio-Rad) with the following parameters: particle bombardment running distance 9 cm, rupture disk pressure 1300 psi and vacuum degree 28 mmHg. After bombardment, the onion tissues were transferred onto new MS agar medium and incubated at 25°C for 12 h in the dark, and the green fluorescence of the cells was observed using a confocal laser scanning microscope (ZEISS LSM 700).

### Transformation of *Arabidopsis* and phenotype observation

The coding fragment of *GhWRKY17* was inserted into the *BamHI/EcoRI* sites of the binary vector pBI121 to generate the *pBI121-GhWRKY17* recombinant plasmid. The recombinant binary vector was transferred into *Agrobacterium tumefaciens* strain LBA4404, and the positive clones were screened with kanamycin (50 mg/ml). *Columbia* ecotype *Arabidopsis thaliana* plants at the initial fruiting period were transformed by floral dipping [54]. The seeds we obtained were called the T_0_ generation. From the beginning of the T_0_ generation, we screened the seeds on 1/2 MS containing kanamycin (50 mg/ml), and PCR was performed to eliminate false positive plants until the T_3_ transgenic homozygous generation. Wild-type (WT) and T_3_ transgenic lines were planted in the greenhouse at 22°C under a 16 h light/8 h dark cycle to observe the natural senescence phenotype. The rosette leaves were harvested for gene expression analysis.

For the ABA treatment, WT and three transgenic lines were germinated on 1/2 MS solid medium for three days. They were then transferred to MS solid medium containing 10 μM ABA for seven days in the vertical position. The phenotype of the seedlings was recorded, and the root length was measured.

All primers used in this paper are listed in S1 Table and were designed using the OLIGO 7 software [55].

## Results

### Identification and characterization of group IIa WRKY genes in *Gossypium hirsutum*

All the candidate *GhWRKY* proteins were identified using HMMER searching and the SMART program. We obtained 239 *GhWRKYs* in upland cotton, and 3 genes with incomplete WRKY domain structures, *GhWRKY27*, *GhWRKY238* and *GhWRKY239* were classified as group IV (S2 Table). Because of the different names of WRKY genes in different publications, we collected the data, performed a similarity comparison and organized the list in S2 Table for easy reference. The *GhWRKYs* were mapped to different *Gossypium hirsutum* genome chromosomes and showed a heterogeneous distribution (S1 Fig). The proteins that clustered with group IIa *AtWRKYs* were considered to be group IIa *GhWRKYs* (S2 Fig). We identified 15 group IIa *GhWRKY* members (S2 and S3 Tables). The 15 group IIa *GhWRKYs* were mainly scattered on chromosomes 5, 6 and 7 and ranged from 140 to 1442 amino acids in protein length. However, 9 genes contained 5 exons and 4 introns, accounting for more than half of the total of 15 group IIa *GhWRKY* genes. Moreover, six genes (*GhWRKY17*, *18*, *20*, *44*, *49 and 155*) were found by Dou *et al*. [5] to have similarity exceeding 97% (Table 1).

**Table 1:** General group IIa *GhWRKY* gene information.

Gene name^a^	Gene ID^b^	Start	End	Protein length	Exon	Intron	Gene name^c^	Accession no.^d^	Identity(%)
*GhWRKY17*	Gh_A05G0483	5264475	5265826	313	5	4	*GhWRKY71*	KF669857	97.44
*GhWRKY18*	Gh_A05G0484	5267711	5268953	252	4	3	*GhWRKY70*	KF669834	99.6
*GhWRKY20*	Gh_A05G1019	10243660	10244007	314	5	4	*GhWRKY73*	KF669835	99.35
*GhWRKY39*	Gh_A06G0917	36697073	36697435	313	5	4	—		—
*GhWRKY44*	Gh_A06G1923	148517	149874	326	4	3	*GhWRKY1*	KF669831	100
*GhWRKY49*	Gh_A07G0261	3249562	3250831	305	5	4	*GhWRKY11*	KF669832	98.69
*GhWRKY50*	Gh_A07G0263	3282938	3298611	1442	12	11	—		—
*GhWRKY140*	Gh_D05G0600	4880919	4882276	313	5	4	—		—
*GhWRKY141*	Gh_D05G0601	4884105	4885375	252	4	3	—		—
*GhWRKY144*	Gh_D05G1137	9746886	9747233	316	5	4	—		—
*GhWRKY155*	Gh_D06G1078	24005778	24006137	312	5	4	*GhWRKY83*	KF669802	99.68
*GhWRKY156*	Gh_D06G1082	24288383	24289803	140	3	2	—		—
*GhWRKY163*	Gh_D06G1966	61082887	61083234	326	4	3	—		
*GhWRKY168*	Gh_D07G0317	3360767	3362036	305	5	4	—		—
*GhWRKY169*	Gh_D07G0318	3381256	3383011	309	5	4	—		—

We name all the *GhWRKY* genes based on their gene IDs in the *Gossypium hirsutum* genome sequence database.

Gene name^a^ shows the group IIa *GhWRKY* genes among all the *GhWRKYs* identified.

Gene ID^b^ is the ID no. in the *Gossypium hirsutum* genome sequence database.

Gene name^c^ and Accession no.^d^ indicate the group IIa *GhWRKY* genes identified by Dou *et al*. [5].

“—” represents no results.

### Phylogenetic tree, intron–exon structure and motif composition in group IIa *GhWRKY* genes

**Fig 1:**
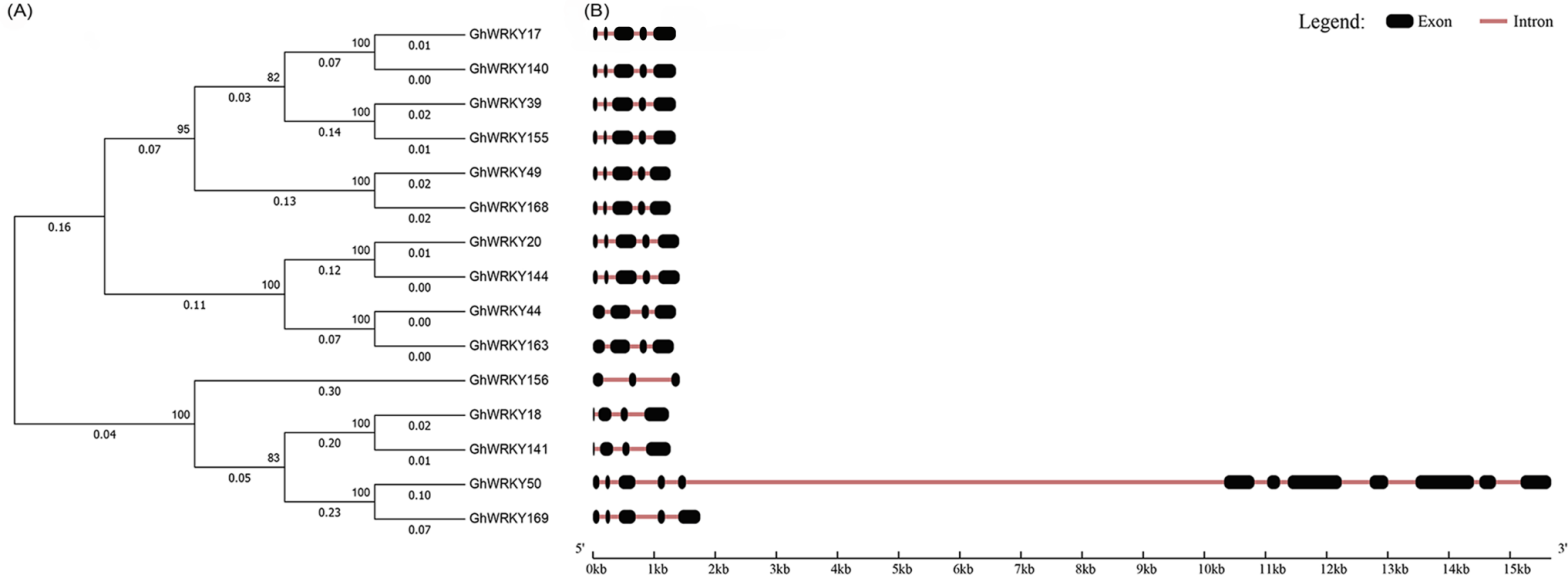
Phylogenetic analysis and exon-intron structures of group IIa *GhWRKY* genes. (A) Phylogenetic analysis among group IIa *GhWRKY* genes. The phylogenetic tree was constructed based on the protein sequences using the MEGA 7 program. The neighbor-joining method was used, and bootstrap analysis was performed with 1000 replications. (B) Exon-intron composition of group IIa *GhWRKY* genes. Exons and introns are represented by black boxes and dark red lines, respectively.

The 15 candidate group IIa *GhWRKYs* were subjected to phylogenetic analysis using the MEGA 7 program with the neighbor-joining method. The phylogenetic tree showed that *GhWRKY17* and *GhWRKY140* were clustered together, followed by *GhWRKY39* and *GhWRKY155*, *GhWRKY49* and *GhWRKY168*, *GhWRKY20* and *GhWRKY144*, *GhWRKY44* and *GhWRKY163*, *GhWRKY18* and *GhWRKY141*, and *GhWRKY50* and *GhWRKY169* (Fig 1A). Among the 15 *GhWRKY* genes, 7 paralogs (*GhWRKY17/140*, *GhWRKY18/141*, *GhWRKY20/144*, *GhWRKY39/155*, *GhWRKY44/163*, *GhWRKY49/168* and *GhWRKY50/169*) were found in the phylogenetic tree (Fig 1A). Among these 7 paralogs, one gene of each paralog comes from the A genome and the other one from the D genome (Table 1). Usually, the criteria for inferring a gene duplication event are that the length of the alignment sequence covers at least 80% of the longest gene and that the similarity of the aligned regions exceeds 70% [56]. By calculating the sequence coverage and similarity, we identified 6 of the 7 paralogs as having undergone gene duplication, while the genes *GhWRKY50* and *GhWRKY169* exhibited less than 70% similarity (18.08%) (Table 2). For convenience, we defined the 6 paralogs as gene pairs here, and a total of 6 gene pairs occurred among the group IIa *GhWRKY* genes. As shown in Fig 1B, the two genes in each gene pair shared a similar intron-exon structure with the same number of introns and exons and similar length at the nucleic acid and amino acid level (Fig 1B). Furthermore, most of the group IIa WRKY genes had 2 to 4 introns, including 1 gene containing 2 introns, 4 genes containing 3 introns and 9 genes containing 4 introns. Interestingly, the gene *GhWRKY50* contained 11 introns (Fig 1B and Table 1).

**Table 2:** The Ka/Ks ratios for duplicate group IIa *GhWRKY* genes.

Paralogous	Identities (%)	Ka	Ks	Ka/Ks	Purifying selection
*GhWRKY17/140*	98.09	0.0173	0.0274	0.6287	YES
*GhWRKY18/141*	98.29	0.0128	0.0315	0.406	YES
*GhWRKY20/144*	97.58	0.9186	0.8633	1.064	NO
*GhWRKY44/163*	98.57	0.0042	0.0481	0.0863	YES
*GhWRKY49/168*	97.6	0.02099	0.0344	0.6066	YES
*GhWRKY39/155*	97.66	—	—	—	—

“—” represents no results.

**Fig 2:**
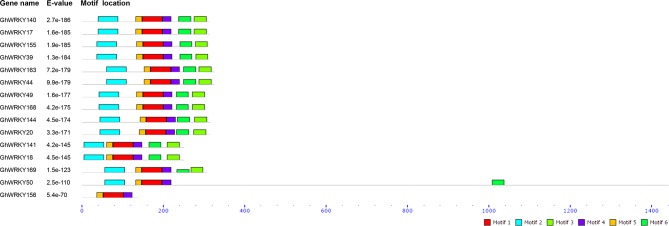
Motif composition in group IIa *GhWRKY* subfamily proteins. Each colored box indicates a different putative motif. The scale plate indicates the protein length. The combined E-value was calculated by the MEME online software.

The MEME software identified six conserved motifs among the group IIa WRKY proteins. The results showed high similarity in a conserved sequence frame within the group IIa WRKY members. All the members contained six motifs except for *GhWRKY50*, which lacked motif 3, and *GhWRKY156*, which lacked motifs 2, 3 and 6 (Fig 2). Moreover, the two genes in each gene pair possessed the same motif composition (Fig 2).

### Ka/Ks analysis

To characterize the evolutionary history of the group IIa *GhWRKY* genes, Ka, Ks and the Ka/Ks ratio were calculated. Apart from the unidentified pair *GhWRKY39/155*, the Ka/Ks ratios of five pairs (*GhWRKY17/140*, *GhWRKY18/141*, *GhWRKY20/144*, *GhWRKY44/163* and *GhWRKY49/168*) were calculated. The similarity of the 5 homologous protein-coding gene pairs exceeded 97% (Table 2). The Ka and Ks values of all 5 gene pairs were lower than 1. For the 4 gene pairs *GhWRKY17/140*, *GhWRKY18/141*, *GhWRKY44/163* and *GhWRKY49/168*, the Ka values were lower than their Ks values, with Ka less than 0.03, and their Ka/Ks ratios were therefore less than 1. However, for the remaining pair, *GhWRKY20/144*, the Ka/Ks ratio was greater than 1 (Table 2).

### Distinct expression profiles of group IIa *GhWRKY* genes in various tissues and at different leaf senescence stages

To further study the function of group IIa *GhWRKYs* in cotton, RNA-Seq data from a public database were used to detect group IIa *GhWRKY* gene expression in twelve tissues and at different leaf senescence stages. According to the RNA-Seq data, no transcript expression was detected for *GhWRKY156*, but the remaining 14 genes showed differential expression levels.

**Fig 3:**
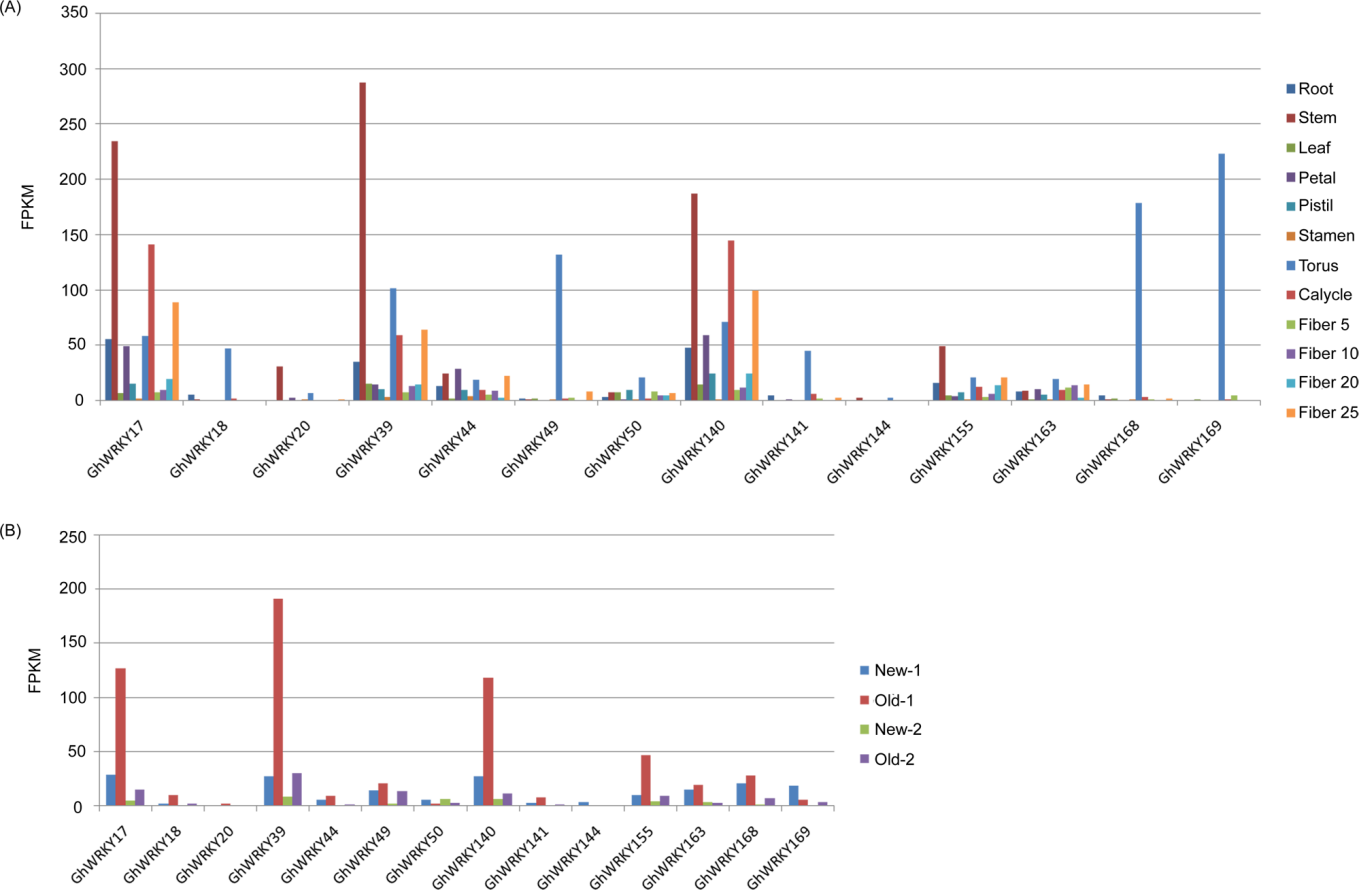
Expression patterns of group IIa *GhWRKY* genes in various tissues and at different leaf senescence stages. (A) Expression patterns of group IIa *GhWRKY* genes in root, stem, leaf, petal, pistil, stamen, torus, calycle, fiber 5, fiber 10, fiber 20 and fiber 25 tissues. Fiber 5, fiber 10, fiber 20 and fiber 25 indicate fiber development stages at 5, 10, 20, and 25 days after anthesis. (B) Expression patterns of group IIa *GhWRKY* genes in leaf senescence. New-1 and old-1 indicate leaves from the three-leaf stage. New-2 and old-2 indicate leaves from the maturation/senescence stages.

As shown in Fig 3A, *GhWRKY17*, *GhWRKY39* and *GhWRKY140* had relatively high expression levels and showed differential expression levels in almost all tissues. The expression of *GhWRKY17* and *GhWRKY140* was the highest in the stem (FPKM>180), followed by the calycle (FPKM>140) and fiber 25 (FPKM>80). The expression of *GhWRKY39* was also the highest in the stem (FPKM>280), but followed by the torus (FPKM>100) and fiber 25 (FPKM>50). However, the remaining genes showed relatively low expression levels overall, with some genes presenting high expression levels in specific tissues. For example, *GhWRKY49*, *GhWRKY168* and *GhWRKY169* were highly expressed specifically in the torus with FPKM values greater than 130 but were hardly expressed in other tissues (Fig 3A).

To detect the gene expression patterns during leaf senescence, we analyzed the RNA-Seq data on new and old leaves from both the three-leaf stage and the maturation/senescence stages. Fig 3B shows that as observed in the tissue expression, the three genes *GhWRKY17*, *GhWRKY39* and *GhWRKY140* also exhibited relatively high expression levels and showed differential expression in new and old leaves. These three genes were specifically highly expressed in old-1 leaves from the three-leaf stage with FPKM values more than 110, and the FPKM value of *GhWRKY39* was near 200. However, the remaining genes in both new and old leaves had FPKM values no greater than 50 (Fig 3B). Taken together, *GhWRKY17*, *GhWRKY39* and *GhWRKY140* may be preferable genes for follow-up studies.

### Tissue-specific and senescence expression patterns of *GhWRKY17*, *GhWRKY39* and *GhWRKY140* analyzed by qRT-PCR

**Fig 4:**
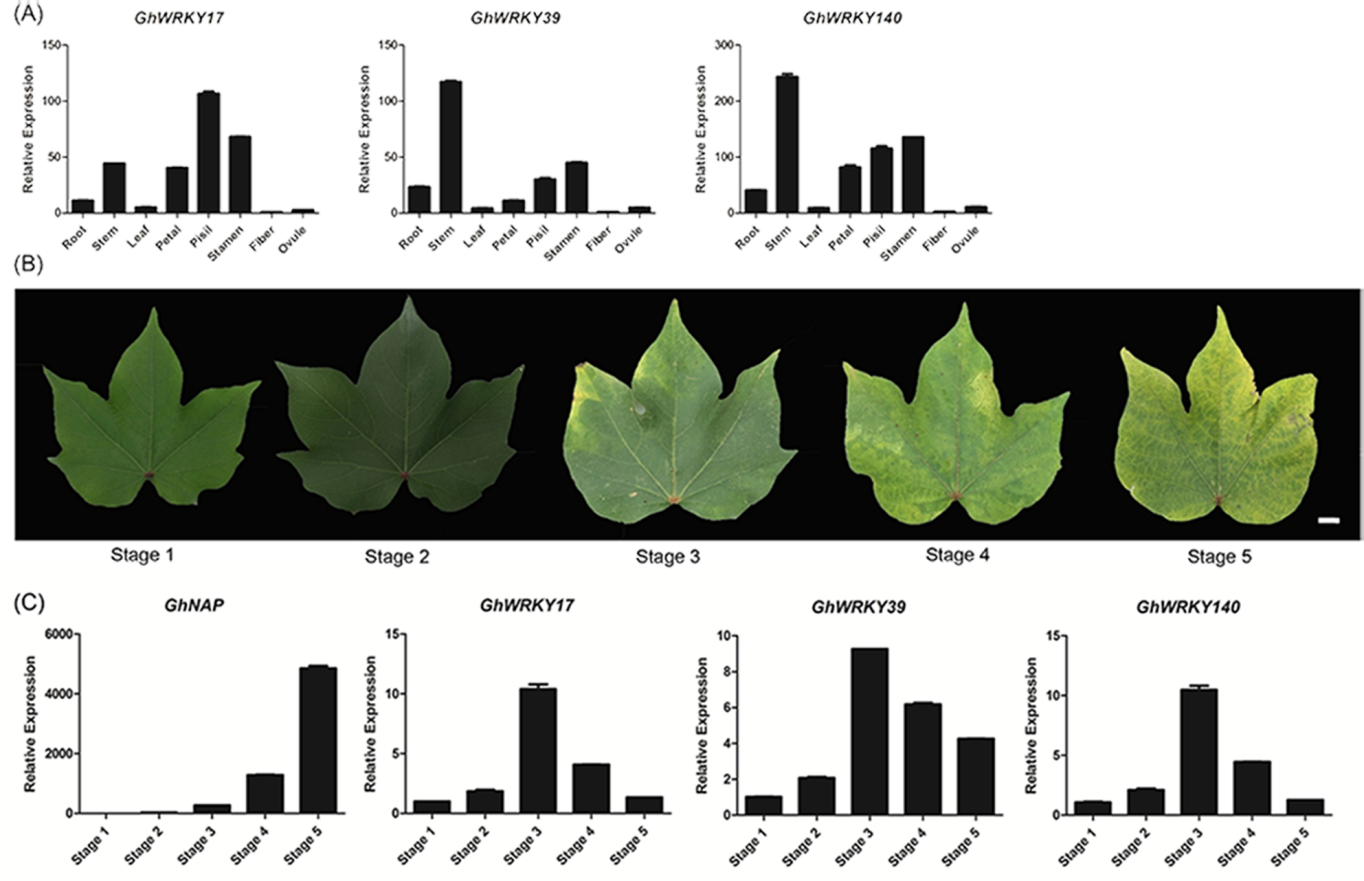
Tissue-specific and senescence expression patterns of *GhWRKY17*, *GhWRKY39* and *GhWRKY140*. (A) Relative expression of *GhWRKY17/39/140* in root, stem, leaf, petal, pistil, stamen, fiber and ovule tissues. The root and stem were sampled from two-week-old seedlings. The leaf, petal, pistil and stamen were sampled at the full flower period. The fiber and ovule were sampled 10 days after anthesis. (B) Five different senescence degrees of true leaves in CCRI74. Stage 1, an expanded new leaf; Stage 2, a mature but non-senescent leaf; Stage 3, a leaf with 25% senescence area; Stage 4, a leaf with 50% senescence area; and Stage 5, a leaf with at least 75% senescence area. (C) Relative expression of *GhNAP* and *GhWRKY17/39/140* in cotton. When qRT-PCR was performed, *GhActin* was used as a reference gene. The data are presented as the means±standard error.

To further discover the functions of group IIa *GhWRKY* genes in different tissues and different stages of the leaf senescence process, according to the expression profiles above, we selected three genes (*GhWRKY17*, *GhWRKY39* and *GhWRKY140*) with high expression abundance and differential expression levels to conduct qRT-PCR using CCRI74 material. The tissue expression results showed that the three genes were highly expressed in the stem and floral organs, and *GhWRKY39* and *GhWRKY140* presented similar expression patterns (Fig 4A).

**Fig 5:**
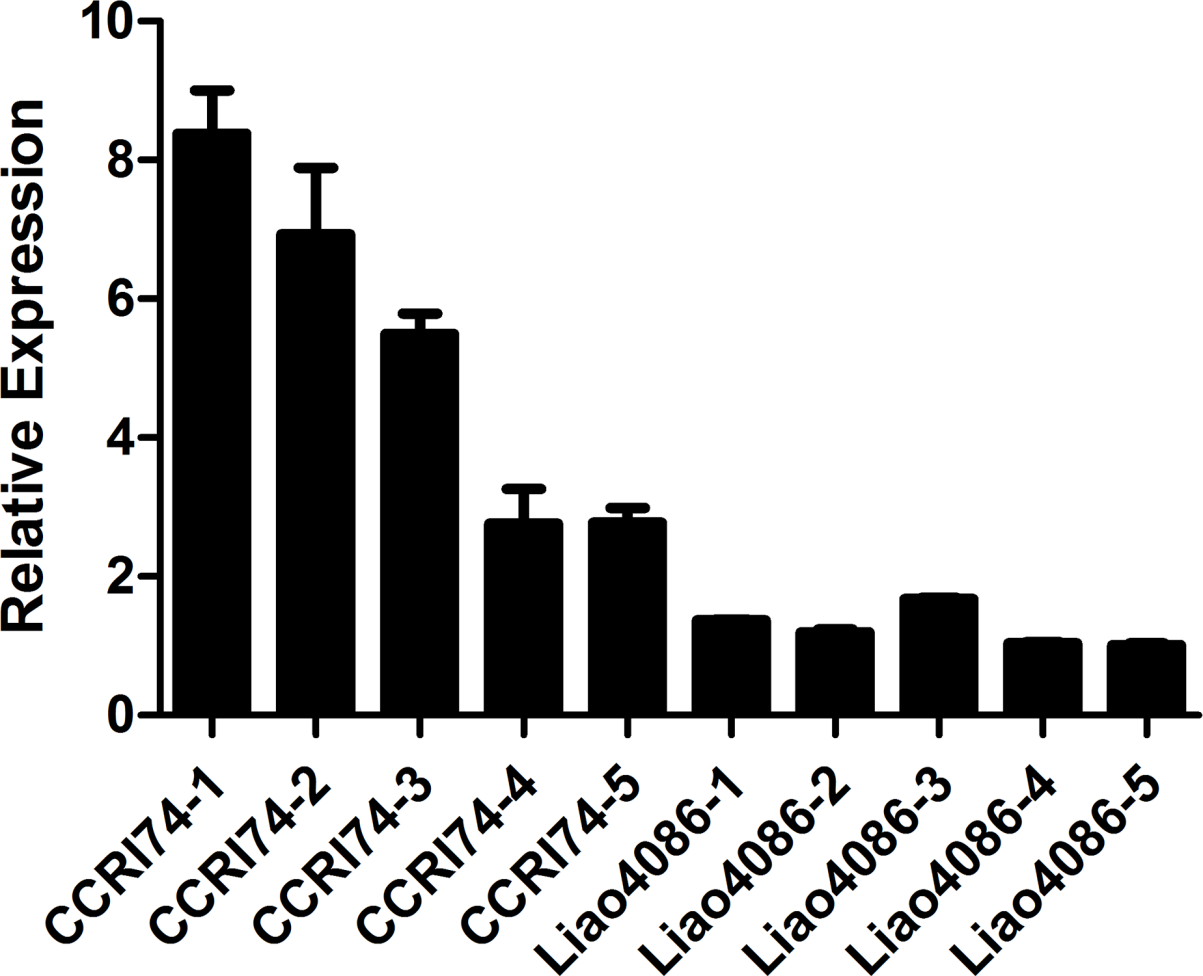
Relative expression of *GhWRKY17* in the early-aging variety CCRI74 and the non-early-aging variety Liao4086 in true leaves at five developmental stages. The fourth leaves from the top of CCRI74 and Liao4086 plants were marked every 10 days from the 80^th^ day after sowing. CCRI 74–1, -2, -3, -4, -5 and Liao 4086–1, -2, -3, -4, -5 represent the true leaves collected 10 days, 20 days, 30 days, 40 days and 50 days after the leaves were marked. The data are presented as the means±standard error. *GhActin* was used as a reference gene.

To determine the involvement of the three genes in leaf senescence, we focused on their expression levels at five development phases of blades with different aging of the leaf area (Fig 4B). *GhNAP* [57] is an up-regulated senescence marker gene in cotton. During leaf development from stage 1 (an expanded new leaf) to stage 5 (a leaf with at least 75% senescence area), the expression level of *GhNAP* was gradually up-regulated, which confirmed the phenotype above (Fig 4B and 4C). However, *GhWRKY17*, *GhWRKY39* and *GhWRKY140* all showed first an increasing and then a descending tendency. They peaked at stage 3, which is the starting point of aging (Fig 4C). Given the similar expression patterns of the three genes, *GhWRKY17* was selected to identify the differential expression at different developmental stages of leaves from the early-aging variety CCRI74 and the non-early-aging variety Liao4086. The results showed that the expression of *GhWRKY17* was higher in CCRI74 than in Liao4086 (Fig 5). As the leaves developed, the expression level of *GhWRKY17* in CCRI74 declined gradually, but little change was observed in Liao4086 (Fig 5). Therefore, the three genes may participate in the initiation of leaf senescence, and *GhWRKY17* plays an important role in leaf senescence.

### Isolation and sequence analysis of *GhWRKY17*

**Fig 6:**
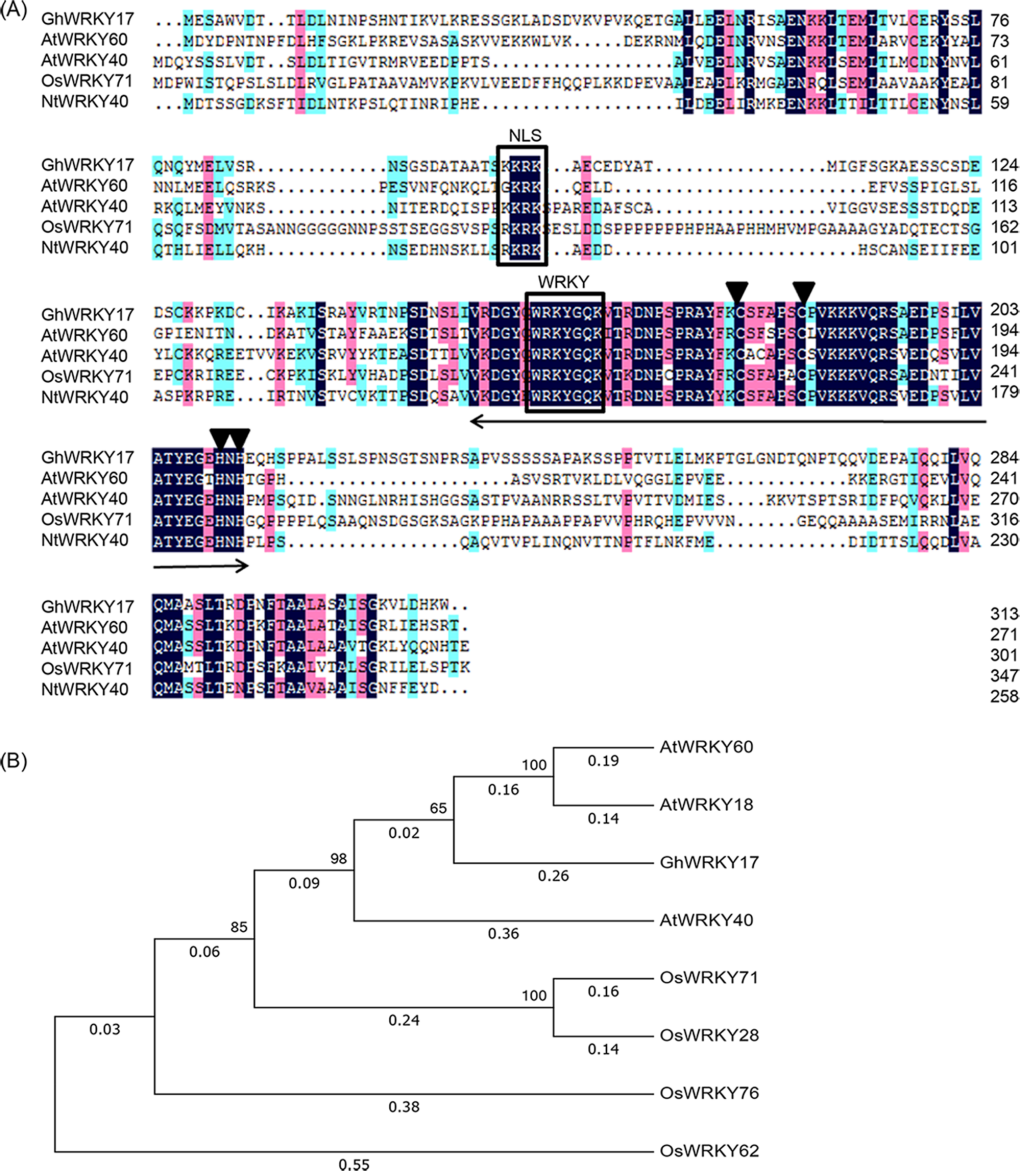
Multiple sequence alignment and phylogenetic analysis of *GhWRKY17*. (A) Multiple sequence alignment of *GhWRKY17* protein with its homologs from different species. The WRKY domain is indicated by a double-headed arrow. The putative NLS and WRKY core sequence are boxed. The zinc finger motif is marked with a downward-pointing triangle. (B) Phylogenetic tree of *GhWRKY17* protein with its homologs from *Arabidopsis thaliana* and *Oryza sativa*. The phylogenetic tree was constructed based on the protein sequences using the MEGA 7 program. The neighbor-joining method was used, and bootstrap analysis was performed with 1000 replications. At, *Arabidopsis thaliana*; Gh, *Gossypium hirsutum*; Nt, *Nicotiana tabacum*; Os, *Oryza sativa*.

We successfully isolated the *GhWRKY17* gene from upland cotton. Sequence analysis revealed that the complete open reading frame (ORF) of *GhWRKY17* was 942 bp in length. A predicted protein with 313 amino acids was encoded by the ORF with a predicted isoelectric point (pI) of 5.08 and molecular weight (Mw) of 77.58 kDa. Multiple sequence alignment analysis of the *GhWRKY17* protein with its homologs *AtWRKY60*, *AtWRKY40*, *OsWRKY71* and *NtWRKY40* revealed that the deduced protein has a nuclear localization signal (NLS) in the N-terminus together with a single WRKY domain in the C-terminus, which contains a conserved WRKYGQK core sequence and a C2H2 zinc finger-like motif (Fig 6A).

To understand the evolutionary relationship, a phylogenetic tree was constructed using the protein sequences of *GhWRKY17* and group IIa *WRKY* genes from other species such as *Arabidopsis* [58] and rice [59]. In the phylogenetic tree, *GhWRKY17* was mainly clustered with *Arabidopsis* genes, and *GhWRKY17* exhibited the highest homology with *AtWRKY18* and *AtWRKY60* (Fig 6B). Therefore, the evolutionary analysis of *GhWRKY17* can provide a reference for its functional study.

### Promoter analysis of *GhWRKY17*

To determine whether *GhWRKY17* is induced by stress and to elucidate the underlying biological mechanism of the gene, a 1500 bp fragment from the upstream region was cloned. *Cis*-acting elements in the promoter region of *GhWRKY17* were predicted using the online *cis*-element prediction software PlantCARE. Many environmental response elements for abiotic stress (anaerobic, heat, drought, and defense elements), hormone stress (ABA, MeJA and gibberellic acid (GA)), light, metabolism and plant development were identified (Table 3). In addition, *GhWRKY17* could be induced by various stresses, including MeJA, ABA, SA, ETH, PEG6000 and NaCl (S3 Fig). Moreover, ABA treatment of transgenic *Arabidopsis* resulted in significantly reduced root length compared with that in the WT (S4 Fig). Our results indicated that *GhWRKY17* might be involved in multiple signaling pathways in plant growth and development and in stress responses.

**Table 3:** The predicted *cis*-acting elements in the promoter of *GhWRKY17*.

Cis-element	Position	Sequence (5'-3')	Function
***Stress-responsive elements***			
ABRE	-927(+),-930(-),-928(+),-931(+)	GGACACGTGGC	Cis-acting element involved in ABA response
ARE	-1137(-),-1431(-),-1254(-)	TGGTTT	Cis-acting regulatory element essential for the anaerobic induction
CGTCA motif	-264(+)	CGTCA	Cis-acting regulatory element involved in MeJA response
HSE	-605(+)	AAAAAATTTC	Cis-acting element involved in heat stress response
MBS	-621(+)	TAACTG	MYB binding site involved in drought-inducibility
P-box	-1440(+)	CCTTTTG	Gibberellin-responsive element
TC-rich repeats	-227(-)	ATTTTCTTCA	Cis-acting element involved in defense and stress response
TGACG motif	-264(-)	TGACG	Cis-acting regulatory element involved in MeJA response
***Light-responsive elements***			
3-AF1 binding site	-35(+)	TAAGAGAGGAA	Light-responsive element
AT1 motif	-543(+)	AATTATTTTTTATT	Part of a light-responsive module
ATCT motif	-417(+)	AATCTAATCC	Part of a conserved DNA module involved in light response
Box 4	-62(+),-628(+),-540(+),-877(-)	ATTAAT	Part of a conserved DNA module involved in light response
G-Box	-45(-),-930(-),-921(+),-940(-)	CACGT(T/G)	Cis-acting regulatory element involved in light response
GATA motif	-38(+),-106(+)	GATAGGA	Part of a light-responsive element
I-box	-1402(-)	AAGATAAGGCT	Part of a light-responsive element
L-box	-1032(-),-1378(-),-1034(-)	TCTCACCTACCAA	Part of a light-responsive element
LAMP-element	-5(-),-1251(+)	CCAAAACCA	Part of a light-responsive element
Box I	-1443(-)	TTTCAAA	Light-responsive element
MNF1	-925(-)	GTGCCC(A/T)(A/T)	Light-responsive element
chs-Unit 1 m1	-1030(-)	ACCTACCACAC	Part of a light-responsive element
***Metabolism and development-related elements***			
O2-site	-254(+),-390(-)	GATGA(C/T)ATGG	Cis-acting regulatory element involved in zein metabolism regulation
Skn-1 motif	-1153(+),-255(-)	GTCAT	Cis-acting regulatory element required for endosperm expression

### *GhWRKY17* is located in the nucleus

**Fig 7:**
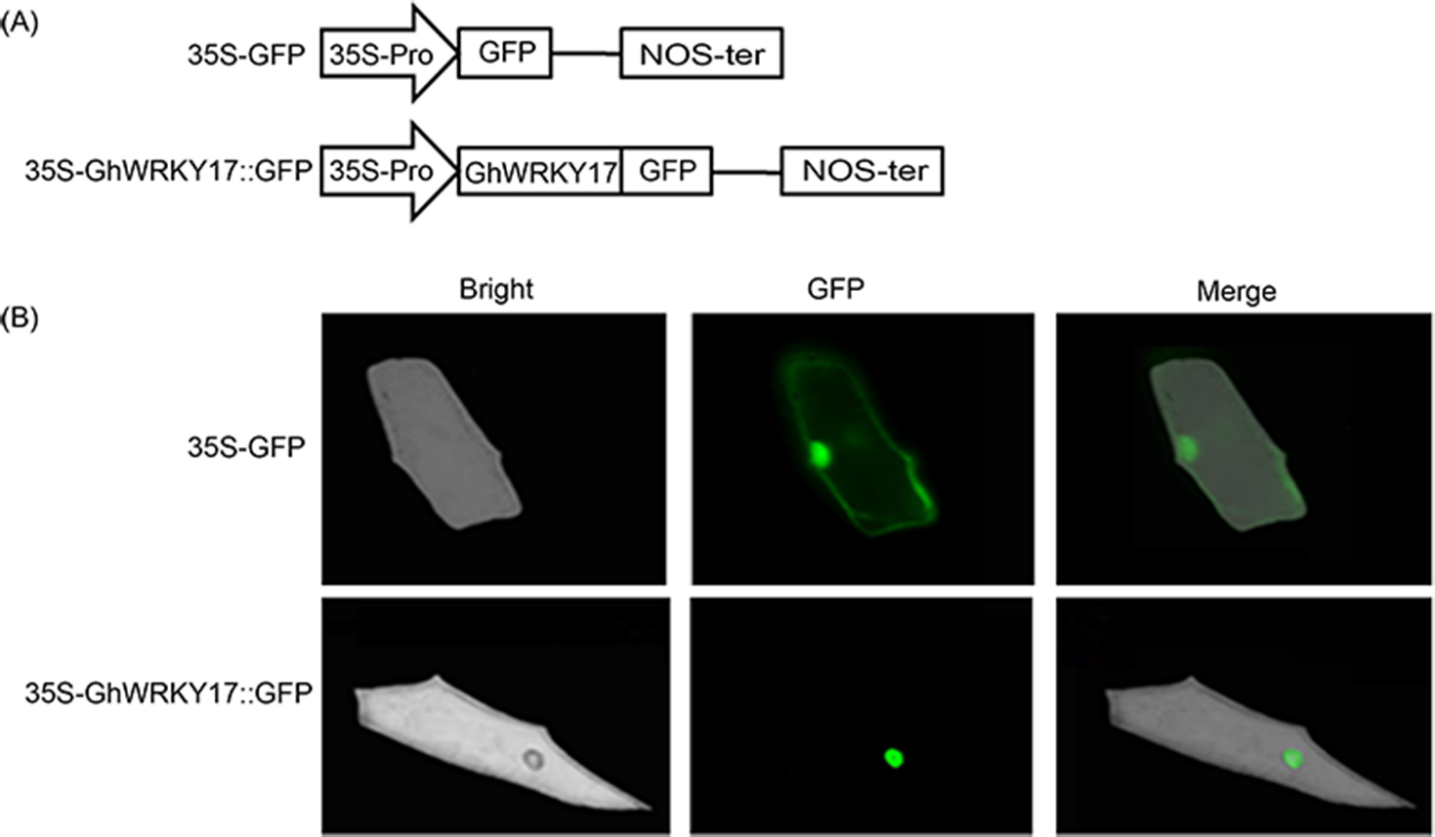
Nuclear localization of *GhWRKY17* in onion epidermal cells. (A) Plasmid sketch of the *35S-GFP* empty vector and *35S-GhWRKY17*::*GFP* fusion construct. *GhWRKY17* was fused to the N-terminus of GFP driven by the *CaMV 35S* promoter. (B) Transient expression of both *35S-GFP* and *35S-GhWRKY17*::*GFP* fusion proteins in onion epidermal cells in bright field (Bright), dim field (GFP), and overlapped field (Merge).

The *GhWRKY17* protein was predicted to be located in the cell nucleus by the online software WoLF PSORT. To determine the subcellular distribution of the *GhWRKY17* protein, the ORF without its termination codon was fused to a GFP gene driven by the *CaMV35S* promoter (Fig 7A). The control *35S-GFP* and experimental *35S-GhWRKY17*:*GFP* plasmids were transformed into cultivated onion epidermal cells using particle bombardment. Microscopic observation showed that green fluorescence in the control was distributed in the nucleus and cytoplasm, but the fusion protein was localized in the nucleus (Fig 7B), indicating that the *GhWRKY17* protein is a nucleoprotein.

### Over-expression of *GhWRKY17* results in precocious leaf senescence

**Fig 8:**
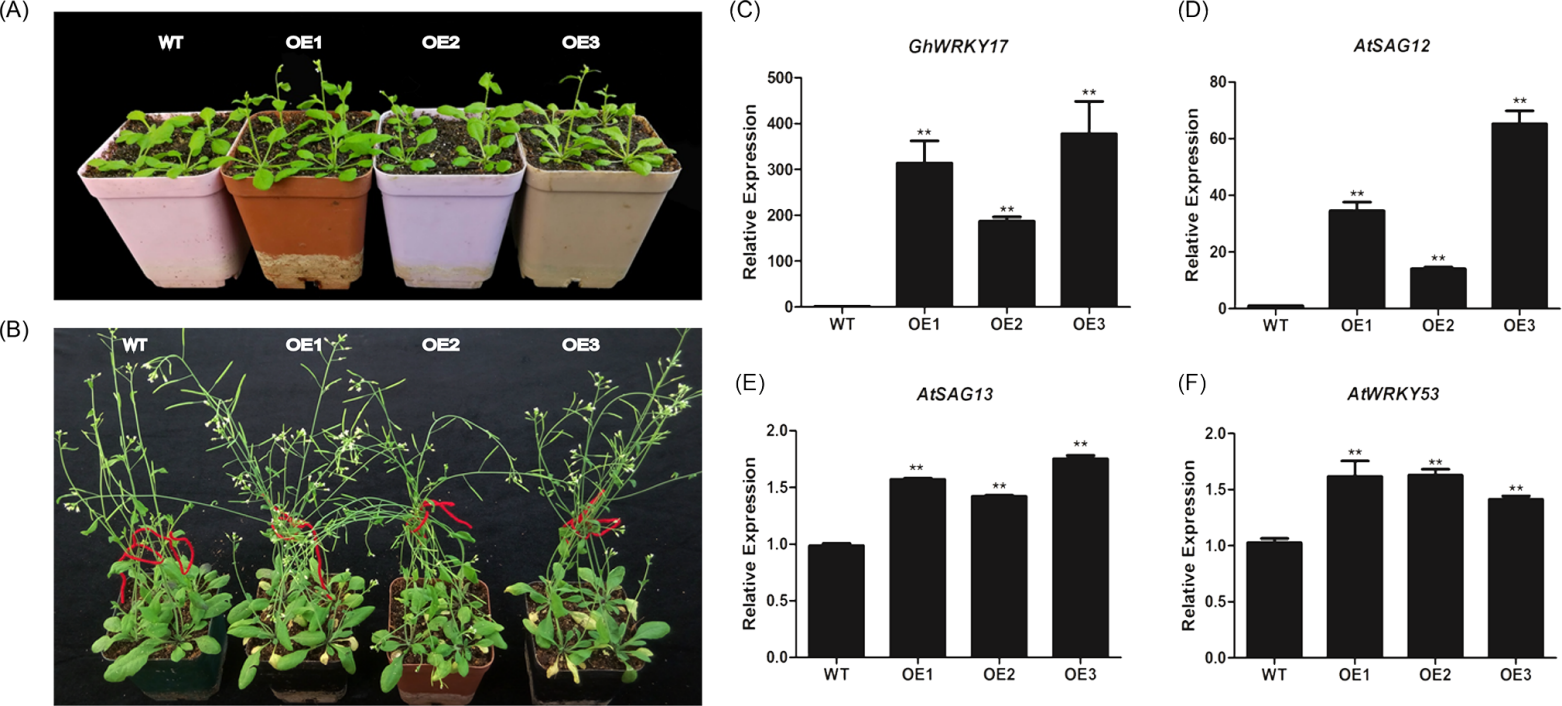
Over-expression of *GhWRKY17* can promote leaf senescence in *Arabidopsis thaliana*. (A) Flowering phenotypes of WT and OE1, OE2 and OE3 transgenic lines. (B) Senescent phenotypes of WT and OE1, OE2 and OE3 transgenic lines. (C) Confirmation of transgenic lines by qRT-PCR. (D-F) Relative expression of the senescence-associated genes *AtSAG12*, *AtSAG13* and *AtWRKY53*. OE1, OE2 and OE3 represent three transgenic lines. When qRT-PCR was performed, *AtUBQ10* was used as a reference gene. The data are presented as the means±standard error. Values significantly different from WT at the 0.01 confidence level.

To assess the function of *GhWRKY17* in the model plant *Arabidopsis*, an expression vector was constructed and transformed into *Arabidopsis* using the floral dipping method [54]. The three transgenic lines were confirmed by qRT-PCR and used to observe the natural growth phenotype (Fig 8A–8C). The transgenic lines showed an early flowering phenotype, while the WT did not (Fig 8A). When the transgenic lines showed an aging phenotype and turned yellow, the WT was still green (Fig 8B). Furthermore, the expression levels of the three senescence marker genes *AtSAG12*, *AtSAG13*, and *AtWRKY53* were significantly higher than in the WT (Fig 8D–8F). Therefore, we concluded that the over-expression of the *GhWRKY17* gene in *Arabidopsis* could result in early senescence.

## Discussion

WRKY TFs are widely involved in the processes of defense, trichome development, plant growth and development and leaf senescence. In this study, we comprehensively analyzed the group IIa WRKY genes in *G*. *hirsutum* using bioinformatics and qRT-PCR analysis. We particularly explored the roles of group IIa WRKY genes in leaf senescence. Thereafter, *GhWRKY17*, one of the group IIa WRKY genes that are differentially expressed in leaf senescence, was isolated and extensively studied. In the functional analysis, the over-expression of *GhWRKY17* in *Arabidopsis* elevated the expression of SAGs and promoted leaf senescence. Our findings extend our knowledge of the functional roles of group IIa WRKY genes in cotton.

The publication of cotton genome sequences for the D genome [60], A genome [61] and AD genome [33,34] provides a basis for the identification and analysis of the group IIa WRKY gene subfamily. Previous studies indentified 7 group IIa WRKY genes in *G*. *raimondii*, 7 in *G*. *arboreum* and 6 in *G*. *hirsutum* [36,37,5]. Here, we identified a total of 15 predicted group IIa *GhWRKY* genes in upland cotton. Among these genes, six (*GhWRKY17*, *18*, *20*, *44*, *49* and *155*) were previously identified by Dou *et al*. [5], and the remaining nine genes (*GhWRKY39*, *50*, *140*, *141*, *144*, *156*, *163*, *168* and *169*) are newly predicted. *G*. *hirsutum* was produced by the interspecific hybridization and polyploidization of *G*. *raimondii* and *G*. *arboretum* [62]. Polyploidization results in whole-genome duplication, and the whole-genome sequencing of cotton revealed massive large-scale gene duplications [63,64,34]. Usually, gene duplication is defined by the length of the alignment sequence covering at least 80% of the longest gene and the similarity of the aligned regions exceeding 70% [56]. The *AtWRKY* gene family was shown to exhibit a certain amount of tandem and segmental duplications [65]. Our results showed 7 paralogs, but only 6 paralogs (*GhWRKY17/140*, *GhWRKY18/141*, *GhWRKY20/144*, *GhWRKY39/155*, *GhWRKY44/163* and *GhWRKY49/168*) appeared to have undergone a high gene duplication event. The 6 paralogs were considered as 6 gene pairs. In these 6 gene pairs, one gene was from At and the other from Dt, and they were located on different chromosomes, which was confirmed to represent fragment duplication [66]. The two genes in each gene pair possessed similar and conserved intron-exon and motif structures, indicating their close evolutionary relationship. Interestingly, although *GhWRKY50* and *GhWRKY169* were clustered together, *GhWRKY50* covered only the front part of *GhWRKY169* and shared a similar structure in the overlap region, which might be explained by the insertion of a long terminal repeat and expansion [61].

The Ka/Ks ratio is as a fairly good indicator for the identification of selective pressure acting on a set of homologous protein-coding genes at the sequence level. As a method of estimating the gene diversity caused by duplication, Ka/Ks>1 implies positive selection, Ka/Ks = 1 implies neutral selection and Ka/Ks<1 implies purifying selection. These values indicate the purifying selection, neutral mutations and beneficial mutations during the evolutionary process [67–69]. The Ka/Ks values of *GhWRKY17/140*, *GhWRKY18/141*, *GhWRKY44/163* and *GhWRKY49/168* were lower than 1, indicating that these 4 gene pairs were under purifying selection and that these genes tended to eliminate deleterious mutations during evolution [70]. The Ka/Ks of *GhWRKY20/144* was greater than 1, suggesting that its evolution at the protein level was accelerating under positive selection.

The publication of a large amount of microarray expression data and expression profiling data provided a basis for the analysis of gene functions. The expression patterns of group IIa WRKY members were preliminarily identified using the expression atlas data for different tissues and different senescence stages [5,34,49]. One gene, *GhWRKY156*, showed no detectable expression in the tissues or different senescent leaves when screening the expression data. The expression level of this gene may be particularly low, or the gene might be expressed only under specific conditions [9]. The expression analysis revealed that *GhWRKY49*, *GhWRKY168* and *GhWRKY169* were highly expressed in the torus, suggesting a potential role in floral development. *GhWRKY17*, *GhWRKY39* and *GhWRKY140* showed relatively high expression levels in both different tissues and during the leaf senescence process. The qRT-PCR results showed that *GhWRKY17*, *GhWRKY39*, and *GhWRKY140* exhibited high expression levels in the flower organs and stem and were also highly expressed during the initial stage of leaf senescence. The WRKY genes are highly expressed in plant organs, indicating that they play an important role in plant growth and development [71]. The group IIa *GhWRKYs* are aging-related genes according to the expression profile analysis [5]. Therefore, we speculated that these three highly expressed genes *GhWRKY17*, *GhWRKY39* and *GhWRKY140* played a significant role in regulating cotton development and leaf senescence. However, more research is needed to clarify the specific functions of these genes.

To gain a greater understanding of the functions of group IIa *GhWRKY* TFs in cotton, the *GhWRKY17* gene was isolated from upland cotton. Several putative stress-related *cis*-regulatory elements were found in the promoter region of *GhWRKY17*. The defense-regulatory elements in the promoter of *CaWRKY1* indicated its function as a molecular player in the plant defense machinery [72]. The stress-induced expression of *GhWRKY17* in cotton and the transgenic *Arabidopsis* phenotype under ABA treatment have proven its potential functions in stress signal pathways. A NLS sequence, “KKRK”, was identified in the protein sequence of *GhWRKY17*. A subcellular localization experiment verified that *GhWRKY17* was localized in the nucleus, which was consistent with previous research on WRKY TFs in cotton [73]. Therefore, *GhWRKY17* may function as a nucleoprotein in signaling pathways and plant regulatory adaptation ability.

Leaf senescence is a complex process that can be affected by environmental factors and phytohormones and involves a decrease in chlorophyll content, macromolecule degradation, nutrient translocation and yield reduction [74–78]. In addition, leaf senescence is a process under the control of regulatory genes [74]. Previous studies have shown that group IIa *WRKY* TFs play an important role in leaf senescence. Based on microarray data and semi-quantitative RT-PCR analysis, *TaWRKY36* was found to be up-regulated during flag leaf senescence in wheat [79]. The expression level of *GhWRKY17* decreased gradually from the beginning of leaf senescence and revealed a higher expression level in the early-aging cotton cultivar CCRI74 than in the non-early-aging cultivar Liao4086, which suggested that *GhWRKY17* might be involved in leaf senescence. *GhWRKY17* was ectopically expressed in *Arabidopsis* to determine its response to senescence. *AtSAG12*, *AtSAG13* and *AtWRKY53* function as positive senescence regulators and have been used as molecular markers to the study the process of leaf senescence in *Arabidopsis* [80,81]. The over-expression of *GhWRKY17* in *Arabidopsis* resulted in a premature senescence phenotype, as confirmed by the higher expression levels of *AtSAG12*, *AtSAG13* and *AtWRKY53* than in the WT. As shown in the phylogenetic tree, *GhWRKY17* shared higher similarity with *AtWRKY18/40/60* than with *OsWRKY28/62/71/76* and shared the highest homology with *AtWRKY18*, suggesting a function similar to that of *AtWRKY18*. The over-expression of *AtWRKY18* in *Arabidopsis* led to delayed senescence [20]. As we can see, *GhWRKY17* and *AtWRKY18* have counteractive effects on the regulation of leaf senescence. Amino acid sequence alignment showed that only 46% similarity between *GhWRKY17* and *AtWRKY18* at the protein level, which might partially explain the difference in function between *GhWRKY17* and *AtWRKY18*. Therefore, understanding the biological function of group IIa *GhWRKY* genes can enrich our knowledge regarding the functions of WRKY genes in crops. Moreover, our study offers guiding significance for future experimental work.

## Supporting information

S1 Fig Chromosomal location of *GhWRKY* genes in the *Gossypium hirsutum* genome. (PDF)

S2 Fig Phylogenetic tree of WRKY proteins of *Gossypium hirsutum* and *Arabidopsis thaliana*. The protein sequences of all *GhWRKYs* and *AtWRKYs* were aligned using Clustal W. The phylogenetic tree was constructed based on the protein sequences using the MEGA 7 program. The maximum likelihood method was used, and bootstrap analysis was performed with 1000 replications.
(PDF)

S3 Fig Expression levels of *GhWRKY17* under various stress conditions. Ten-day-old healthy and uniform seedlings were irrigated with 15% PEG6000 (A), 200 mM NaCl (B), and sprayed with 100 μM MeJA (C), 200 μM ABA (D), 2 mM SA (E) and 0.5 mM ETH (F). The total RNA was extracted from the samples at 0 h, 2 h, 4 h, 8 h and 12 h after stress treatments. *GhActin* was used as an internal reference. The data are presented as the means±standard error. The bars represent the standard error.
(PDF)

S4 Fig Over-expression of *GhWRKY17* led to susceptibility to ABA treatment in *Arabidopsis*. Three-day-old seedlings grown on 1/2 MS medium were transferred to new MS medium containing 10 μM ABA for seven days. (A) Phenotypic characteristics of WT and transgenic plants under 10 μM ABA treatment for seven days. (B) Root length of seedlings grown on MS medium containing 10 μM ABA for seven days. The data are presented as the means±standard error. The bars represent standard error. Values significantly different from WT at the 0.01 confidence level.
(PDF)

S1 Table Primers used in this study. (DOCX)

S2 Table General information on WRKY gene family in cotton. a: We named all the *GhWRKYs* based on their gene IDs in the genome sequence database. b: Subgroups were divided according to the results of evolutionary analysis. The *GhWRKY* proteins that clustered with *AtWRKY* proteins were considered the same subfamily. c: WRKY genes identified by Cai *et al*. [37], using the sequence information of Paterson *et al*. [82]. d: WRKY genes identified by Cai *et al*. [37], using the sequence information of Wang *et al*. [60]. e: WRKY genes identified by Dou *et al*. [5]. “—” represents no results.
(DOCX)

S3 Table The number of different subfamilies of the *GhWRKY* gene. (DOCX)
