# Supplementary figures and images for: Identification of the group IIa WRKY subfamily and the functional analysis of GhWRKY17 in upland cotton (Gossypium hirsutum L.)
Source: PLoS One. 2018 Jan 25;13(1):e0191681. doi: 10.1371/journal.pone.0191681 (PMC5784973; doi:10.1371/journal.pone.0191681)

A01

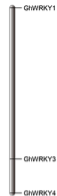

A02

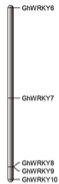

A03

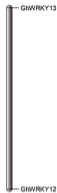

A04

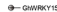

A05

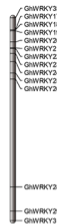

A06

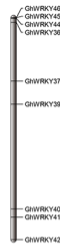

A07

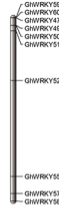

A08

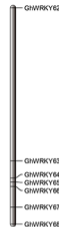

A09

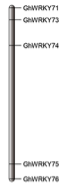

A10

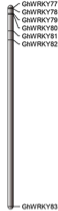

A11

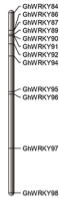

A12

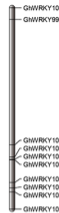

A13

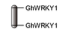

D01

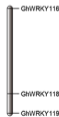

D02

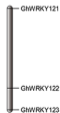

D03

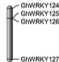

D04

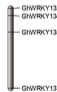

D05

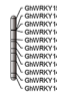

D06

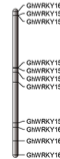

D07

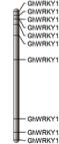

D08

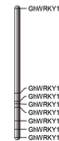

D09

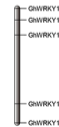

D10

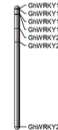

D11

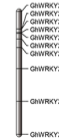

D12

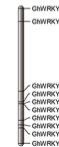

D13

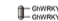

Supplement: S1 Fig — (PDF) [file pone.0191681.s001.pdf]

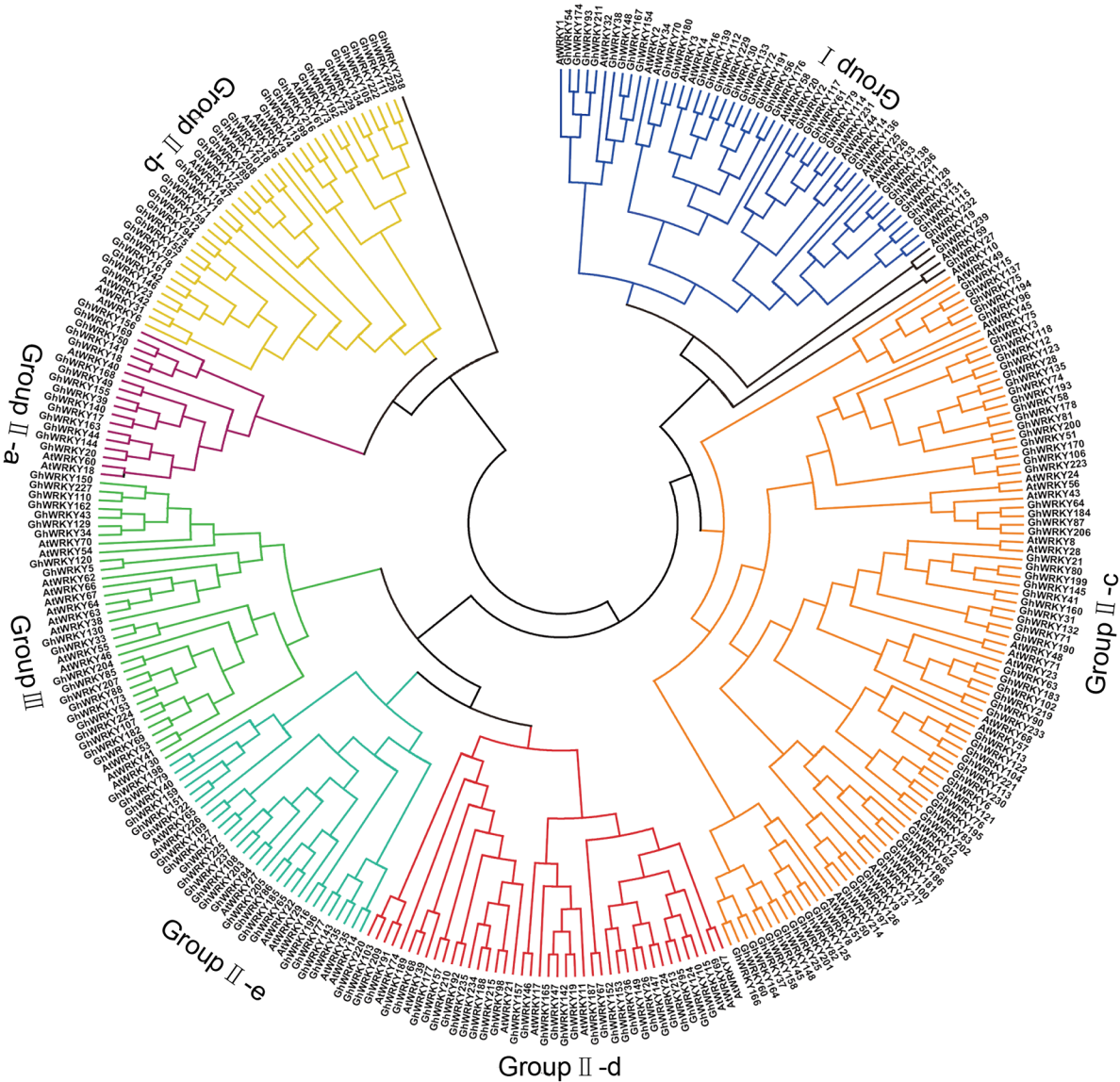

Supplement: S2 Fig — The protein sequences of all GhWRKYs and AtWRKYs were aligned using Clustal W. The phylogenetic tree was constructed based on the protein sequences using the MEGA 7 program. The maximum likelihood method was used, and bootstrap analysis was performed with 1000 replications. (PDF) [file pone.0191681.s002.pdf]

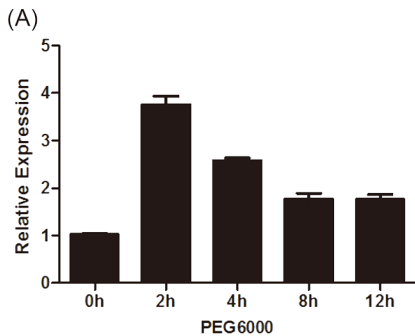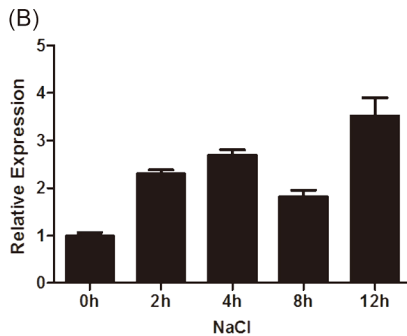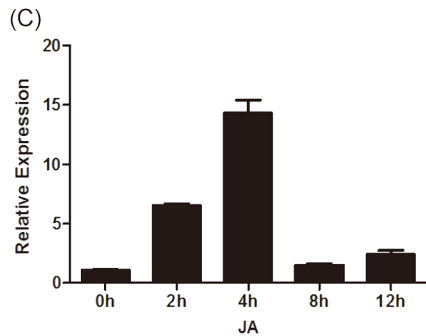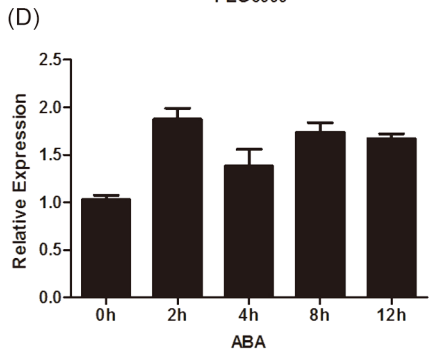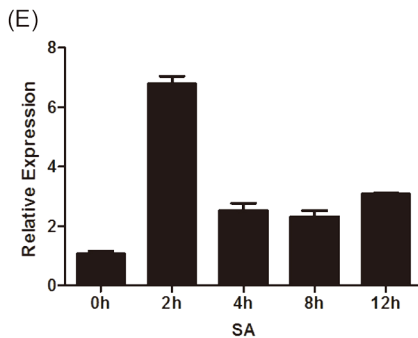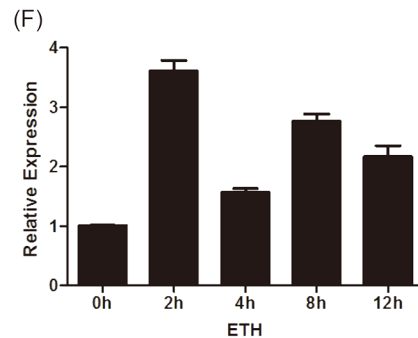

Supplement: S3 Fig — Ten-day-old healthy and uniform seedlings were irrigated with 15% PEG6000 (A), 200 mM NaCl (B), and sprayed with 100 μM MeJA (C), 200 μM ABA (D), 2 mM SA (E) and 0.5 mM ETH (F). The total RNA was extracted from the samples at 0 h, 2 h, 4 h, 8 h and 12 h after stress treatments. GhActin was used as an internal reference. The data are presented as the means±standard error. The bars represent the standard error. (PDF) [file pone.0191681.s003.pdf]

(A) Control 10 $\mu$ M ABA (B)

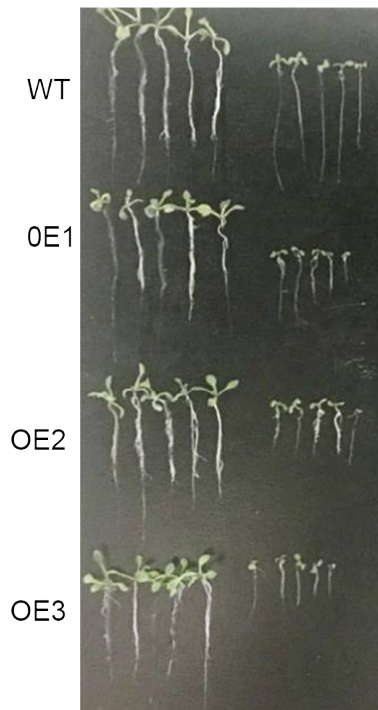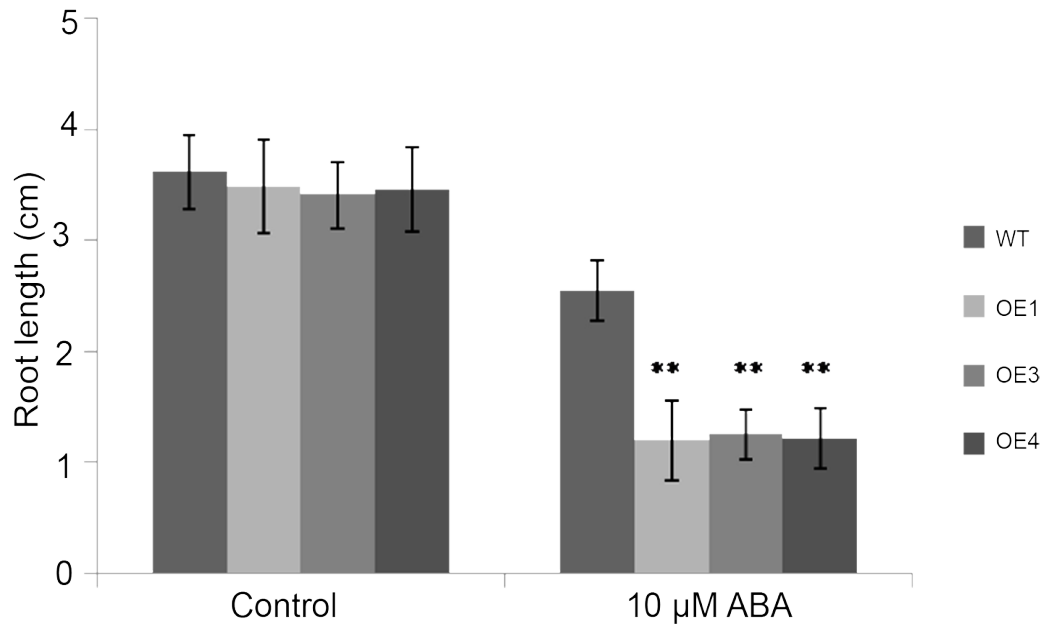

Supplement: S4 Fig — Three-day-old seedlings grown on 1/2 MS medium were transferred to new MS medium containing 10 μM ABA for seven days. (A) Phenotypic characteristics of WT and transgenic plants under 10 μM ABA treatment for seven days. (B) Root length of seedlings grown on MS medium containing 10 μM ABA for seven days. The data are presented as the means±standard error. The bars represent standard error. Values significantly different from WT at the 0.01 confidence level. (PDF) [file pone.0191681.s004.pdf]
