# Supplementary material for: Identification of the group IIa WRKY subfamily and the functional analysis of GhWRKY17 in upland cotton (Gossypium hirsutum L.)
Source: PLoS One. 2018 Jan 25;13(1):e0191681. doi: 10.1371/journal.pone.0191681 (PMC5784973; doi:10.1371/journal.pone.0191681)
Supplement: S1 Table — (DOCX) [file pone.0191681.s005.docx]

| **Primer name** | **Sequence ( 5′- 3′)** | **Description** |
| --- | --- | --- |
| QRT-*GhWRKY17*-F | CAGGAACTCGGGAAGTGATGC | qRT-PCR primers for *GhWRKY17* |
| QRT-*GhWRKY17*-R | GCGCACGTAAGCCCTTGAAA |  |
| QRT-*GhWRKY39*-F | GAGAAAAGCTGAGTGGGAGGA | qRT-PCR primers for *GhWRKY39* |
| QRT-*GhWRKY39*-R | TGCGGACTTGAACCCTAGAAA |  |
| QRT-*GhWRKY140*-F | AAAGCCGAGTGTGAGGATTATGT | qRT-PCR primers for *GhWRKY140* |
| QRT-*GhWRKY140*-R | CTTTGATGCAGTCCTTAGGCTTT |  |
| QRT-*GhNAP*-F | GCCCCAATTCACATGACACAGT | SAGs primers in cotton |
| QRT-*GhNAP*-R | TCTCAACATGGTCACCTGTGGT |  |
| QRT-*AtSAG12*-F | TCCAATTCTATTCGTCTGGTGTGT | SAGs primers in *Arabidopsis thaliana* |
| QRT-*AtSAG12*-R | CCACTTTCTCCCCATTTTGTTC |  |
| QRT-*AtSAG13*-F | GTGCCAGAGACGAAACTC | SAGs primers in *Arabidopsis thaliana* |
| QRT-*AtSAG13*-R | GCTGTAAACTCTGTGGTC |  |
| QRT-*AtWRKY53*-F | CAGACGGGGATGCTACGG | SAGs primers in *Arabidopsis thaliana* |
| QRT-*AtWRKY53*-R | GGCGAGGCTAATGGTGGT |  |
| QRT-*GhActin*-F | ATCCTCCGTCTTGACCTTG | Reference gene in cotton |
| QRT-*GhActin*-R | TGTCCGTCAGGCAACTCAT |  |
| QRT-*AtUBQ10*-F | AGATCCAGGACAAGGAAGGTATTC | Reference gene in *Arabidopsis thaliana* |
| QRT-*AtUBQ10*-R | CGCAGGACCAAGTGAAGAGTAG |  |
| *GhWRKY17*-BamHI-F | CGCGGATCCTCTTTGCTTCTTCTTTTCTTG | Over expression vector construction |
| *GhWRKY17*-EcoRI-R | CCGGAATTCTTCTCCCTAAGTATGTTG |  |
| P17-F | GTTTGTGGTTAAAGCGTCG | Promoter cloning |
| P17-R | CACCGAAATTACACTATAGAACAA |  |
| pBFP17-XbaI-F | CTAGTCTAGAATGGAATCGGCTTGGGTGGAT | Subcellular localization |
| pBFP17-SpeI-R | CTAGACTAGTCCACTTGTGATCTAGAACTTTTCC |  |
| M13-F | TGTAAAACGACGGCCAGT | For the detection of positive clones |
| 35S | GACGCACAATCCCACTATCC | For the detection of positive clones |
