# Supplementary material for: Identification of the group IIa WRKY subfamily and the functional analysis of GhWRKY17 in upland cotton (Gossypium hirsutum L.)
Source: PLoS One. 2018 Jan 25;13(1):e0191681. doi: 10.1371/journal.pone.0191681 (PMC5784973; doi:10.1371/journal.pone.0191681)
Supplement: S2 Table — a: We named all the GhWRKYs based on their gene IDs in the genome sequence database. b: Subgroups were divided according to the results of evolutionary analysis. The GhWRKY proteins that clustered with AtWRKY proteins were considered the same subfamily. c: WRKY genes identified by Cai et al. [37], using the sequence information of Paterson et al. [82]. d: WRKY genes identified by Cai et al. [37], using the sequence information of Wang et al. [60]. e: WRKY genes identified by Dou et al. [5]. “—” represents no results. (DOCX) [file pone.0191681.s006.docx]

| **My work** | | | **Paterson et al.^c^** | | **Wang et al.^d^** | | **Dou et al.^e^** | | | |
| --- | --- | --- | --- | --- | --- | --- | --- | --- | --- | --- |
| **Gossypium hirsutum** | | | **Gossypium raimondii** | | **Gossypium raimondii** | | **Gossypium raimondii** | | **Gossypium hirsutum** | |
| **Gene^a^** | **ID** | **Group^b^** | **Gene** | **ID** | **Gene** | **ID** | **Gene** | **ID** | **Gene** | **Accession NO.** |
| *GhWRKY1* | Gh_A01G0639 | II-b | — | — | — | — | — | — | — | — |
| *GhWRKY2* | Gh_A01G0681 | I | — | — | — | — | — | — | — | — |
| *GhWRKY3* | Gh_A01G1273 | II-c | — | — | — | — | — | — | — | — |
| *GhWRKY4* | Gh_A01G1477 | II-b | — | — | — | — | — | — | — | — |
| *GhWRKY5* | Gh_A02G0029 | III | — | — | — | — | — | — | — | — |
| *GhWRKY6* | Gh_A02G0541 | II-c | — | — | — | — | — | — | — | — |
| *GhWRKY7* | Gh_A02G1042 | II-e | — | — | — | — | — | — | — | — |
| *GhWRKY8* | Gh_A02G1301 | II-c | — | — | — | — | — | — | — | — |
| *GhWRKY9* | Gh_A02G1304 | II-c | — | — | — | — | — | — | — | — |
| *GhWRKY10* | Gh_A02G1488 | II-d | — | — | — | — | — | — | — | — |
| *GhWRKY11* | Gh_A03G0210 | I | — | — | — | — | — | — | — | — |
| *GhWRKY12* | Gh_A03G1081 | II-c | — | — | — | — | — | — | — | — |
| *GhWRKY13* | Gh_A03G2109 | II-c | — | — | — | — | — | — | — | — |
| *GhWRKY14* | Gh_A04G0658 | I | — | — | — | — | — | — | — | — |
| *GhWRKY15* | Gh_A04G0709 | II-c | — | — | — | — | — | — | — | — |
| *GhWRKY16* | Gh_A04G1269 | I | — | — | — | — | — | — | — | — |
| *GhWRKY17* | Gh_A05G0483 | II-a | — | — | — | — | — | — | — | — |
| *GhWRKY18* | Gh_A05G0484 | II-a | — | — | — | — | — | — | — | — |
| *GhWRKY19* | Gh_A05G0528 | II-d | — | — | — | — | — | — | — | — |
| *GhWRKY20* | Gh_A05G1019 | II-a | — | — | — | — | — | — | — | — |
| *GhWRKY21* | Gh_A05G1264 | II-c | — | — | — | — | — | — | — | — |
| *GhWRKY22* | Gh_A05G1287 | II-e | — | — | — | — | — | — | — | — |
| *GhWRKY23* | Gh_A05G1472 | II-b | — | — | — | — | — | — | — | — |
| *GhWRKY24* | Gh_A05G1774 | II-d | — | — | — | — | — | — | — | — |
| *GhWRKY25* | Gh_A05G2091 | II-c | — | — | — | — | — | — | — | — |
| *GhWRKY26* | Gh_A05G2237 | II-d | — | — | — | — | — | — | — | — |
| *GhWRKY27* | Gh_A05G2378 | Ⅳ | — | — | — | — | — | — | — | — |
| *GhWRKY28* | Gh_A05G2938 | II-c | — | — | — | — | — | — | — | — |
| *GhWRKY29* | Gh_A05G3158 | II-b | — | — | — | — | — | — | — | — |
| *GhWRKY30* | Gh_A05G3186 | I | — | — | — | — | — | — | — | — |
| *GhWRKY31* | Gh_A05G3283 | II-c | — | — | — | — | — | — | — | — |
| *GhWRKY32* | Gh_A05G3382 | I | — | — | — | — | — | — | — | — |
| *GhWRKY33* | Gh_A05G3498 | III | — | — | — | — | — | — | — | — |
| *GhWRKY34* | Gh_A05G3499 | III | — | — | — | — | — | — | — | — |
| *GhWRKY35* | Gh_A05G3887 | II-e | — | — | — | — | — | — | — | — |
| *GhWRKY36* | Gh_A06G0179 | II-d | — | — | — | — | — | — | — | — |
| *GhWRKY37* | Gh_A06G0764 | II-c | — | — | — | — | — | — | — | — |
| *GhWRKY38* | Gh_A06G0879 | I | — | — | — | — | — | — | — | — |
| *GhWRKY39* | Gh_A06G0917 | II-a | — | — | — | — | — | — | — | — |
| *GhWRKY40* | Gh_A06G1167 | II-e | — | — | — | — | — | — | — | — |
| *GhWRKY41* | Gh_A06G1188 | II-c | — | — | — | — | — | — | — | — |
| *GhWRKY42* | Gh_A06G1322 | II-b | — | — | — | — | — | — | — | — |
| *GhWRKY43* | Gh_A06G1585 | III | — | — | — | — | — | — | — | — |
| *GhWRKY44* | Gh_A06G1923 | II-a | — | — | — | — | — | — | — | — |
| *GhWRKY45* | Gh_A06G2115 | II-c | — | — | — | — | — | — | — | — |
| *GhWRKY46* | Gh_A06G2118 | II-d | — | — | — | — | — | — | — | — |
| *GhWRKY47* | Gh_A07G0017 | II-d | — | — | — | — | — | — | — | — |
| *GhWRKY48* | Gh_A07G0212 | I | — | — | — | — | — | — | — | — |
| *GhWRKY49* | Gh_A07G0261 | II-a | — | — | — | — | — | — | — | — |
| *GhWRKY50* | Gh_A07G0263 | II-a | — | — | — | — | — | — | — | — |
| *GhWRKY51* | Gh_A07G0441 | II-c | — | — | — | — | — | — | — | — |
| *GhWRKY52* | Gh_A07G1198 | II-b | — | — | — | — | — | — | — | — |
| *GhWRKY53* | Gh_A07G1274 | III | — | — | — | — | — | — | — | — |
| *GhWRKY54* | Gh_A07G1524 | I | — | — | — | — | — | — | — | — |
| *GhWRKY55* | Gh_A07G1665 | II-b | — | — | — | — | — | — | — | — |
| *GhWRKY56* | Gh_A07G1892 | I | — | — | — | — | — | — | — | — |
| *GhWRKY57* | Gh_A07G1912 | II-d | — | — | — | — | — | — | — | — |
| *GhWRKY58* | Gh_A07G2119 | II-c | — | — | — | — | — | — | — | — |
| *GhWRKY59* | Gh_A07G2274 | II-b | — | — | — | — | — | — | — | — |
| *GhWRKY60* | Gh_A07G2317 | II-c | — | — | — | — | — | — | — | — |
| *GhWRKY61* | Gh_A08G0212 | I | — | — | — | — | — | — | — | — |
| *GhWRKY62* | Gh_A08G0526 | II-c | — | — | — | — | — | — | — | — |
| *GhWRKY63* | Gh_A08G1051 | II-c | — | — | — | — | — | — | — | — |
| *GhWRKY64* | Gh_A08G1141 | II-c | — | — | — | — | — | — | — | — |
| *GhWRKY65* | Gh_A08G1191 | II-e | — | — | — | — | — | — | — | — |
| *GhWRKY66* | Gh_A08G1256 | II-c | — | — | — | — | — | — | — | — |
| *GhWRKY67* | Gh_A08G1540 | II-d | — | — | — | — | — | — | — | — |
| *GhWRKY68* | Gh_A08G1914 | II-d | — | — | — | — | — | — | — | — |
| *GhWRKY69* | Gh_A08G2417 | III | — | — | — | — | — | — | — | — |
| *GhWRKY70* | Gh_A08G2488 | I | — | — | — | — | — | — | — | — |
| *GhWRKY71* | Gh_A09G0070 | II-c | — | — | — | — | — | — | — | — |
| *GhWRKY72* | Gh_A09G0118 | I | — | — | — | — | — | — | — | — |
| *GhWRKY73* | Gh_A09G0225 | II-b | — | — | — | — | — | — | — | — |
| *GhWRKY74* | Gh_A09G0361 | II-c | — | — | — | — | — | — | — | — |
| *GhWRKY75* | Gh_A09G1447 | II-c | — | — | — | — | — | — | — | — |
| *GhWRKY76* | Gh_A09G2126 | II-c | — | — | — | — | — | — | — | — |
| *GhWRKY77* | Gh_A10G0054 | II-e | — | — | — | — | — | — | — | — |
| *GhWRKY78* | Gh_A10G0113 | II-b | — | — | — | — | — | — | — | — |
| *GhWRKY79* | Gh_A10G0323 | II-e | — | — | — | — | — | — | — | — |
| *GhWRKY80* | Gh_A10G0359 | II-c | — | — | — | — | — | — | — | — |
| *GhWRKY81* | Gh_A10G0638 | II-c | — | — | — | — | — | — | — | — |
| *GhWRKY82* | Gh_A10G0753 | II-c | — | — | — | — | — | — | — | — |
| *GhWRKY83* | Gh_A10G1549 | II-c | — | — | — | — | — | — | — | — |
| *GhWRKY84* | Gh_A11G0103 | II-e | — | — | — | — | — | — | — | — |
| *GhWRKY85* | Gh_A11G0118 | III | — | — | — | — | — | — | — | — |
| *GhWRKY86* | Gh_A11G0833 | II-e | — | — | — | — | — | — | — | — |
| *GhWRKY87* | Gh_A11G0834 | II-c | — | — | — | — | — | — | — | — |
| *GhWRKY88* | Gh_A11G0868 | III | — | — | — | — | — | — | — | — |
| *GhWRKY89* | Gh_A11G0954 | II-b | — | — | — | — | — | — | — | — |
| *GhWRKY90* | Gh_A11G0984 | II-c | — | — | — | — | — | — | — | — |
| *GhWRKY91* | Gh_A11G0997 | II-d | — | — | — | — | — | — | — | — |
| *GhWRKY92* | Gh_A11G1172 | II-d | — | — | — | — | — | — | — | — |
| *GhWRKY93* | Gh_A11G1391 | I | — | — | — | — | — | — | — | — |
| *GhWRKY94* | Gh_A11G1432 | II-b | — | — | — | — | — | — | — | — |
| *GhWRKY95* | Gh_A11G1801 | II-d | — | — | — | — | — | — | — | — |
| *GhWRKY96* | Gh_A11G1811 | II-c | — | — | — | — | — | — | — | — |
| *GhWRKY97* | Gh_A11G2016 | II-c | — | — | — | — | — | — | — | — |
| *GhWRKY98* | Gh_A11G2258 | II-d | — | — | — | — | — | — | — | — |
| *GhWRKY99* | Gh_A12G0294 | II-b | — | — | — | — | — | — | — | — |
| *GhWRKY100* | Gh_A12G0887 | II-c | — | — | — | — | — | — | — | — |
| *GhWRKY101* | Gh_A12G1084 | II-b | — | — | — | — | — | — | — | — |
| *GhWRKY102* | Gh_A12G1116 | II-c | — | — | — | — | — | — | — | — |
| *GhWRKY103* | Gh_A12G1124 | II-d | *WRKY105* | Gorai.008G138900.1 | *WRKY105* | gene_10027229 | GrWRKY65 | Cotton_D_gene_10027229 | *GhWRKY65* | KF669789 |
| *GhWRKY104* | Gh_A12G1488 | II-c | — | — | — | — | — | — | — | — |
| *GhWRKY105* | Gh_A12G1594 | II-b | — | — | — | — | — | — | — | — |
| *GhWRKY106* | Gh_A12G1670 | II-c | *WRKY109* | Gorai.008G201000.1 | — | — | *GrWRKY64* | Cotton_D_gene_10029945 | *GhWRKY64* | KF669837 |
| *GhWRKY107* | Gh_A12G2121 | III | — | — | — | — | — | — | — | — |
| *GhWRKY108* | Gh_A12G2292 | II-e | — | — | — | — | — | — | — | — |
| *GhWRKY109* | Gh_A12G2671 | II-e | — | — | — | — | — | — | — | — |
| *GhWRKY110* | Gh_A13G0059 | III | — | — | — | — | — | — | — | — |
| *GhWRKY111* | Gh_A13G0630 | II-b | — | — | — | — | — | — | — | — |
| *GhWRKY112* | Gh_A13G0696 | I | — | — | — | — | — | — | — | — |
| *GhWRKY113* | Gh_A13G0715 | II-c | — | — | — | — | — | — | — | — |
| *GhWRKY114* | Gh_A13G1138 | I | — | — | — | — | — | — | — | — |
| *GhWRKY115* | Gh_A13G1330 | I | — | — | — | — | — | — | — | — |
| *GhWRKY116* | Gh_D01G0657 | II-b | *WRKY001* | Gorai.002G091300.1 | WRKY001 | gene_10024898 | *GrWRKY16* | Cotton_D_gene_10024898 | *GhWRKY16* | KF669824 |
| *GhWRKY117* | Gh_D01G0702 | I | *WRKY002* | Gorai.002G096200.1 | *WRKY002* | gene_10024943 | *GrWRKY17* | Cotton_D_gene_10024943 | *GhWRKY17* | KF669761 |
| *GhWRKY118* | Gh_D01G1493 | II-c | *WRKY003* | Gorai.002G181600.1 | *WRKY003* | gene_10020058 | *GrWRKY15* | Cotton_D_gene_10020058 | *GhWRKY15* | KF669833 |
| *GhWRKY119* | Gh_D01G1714 | II-b | *WRKY004* | Gorai.002G207600.1 | — | — | *GrWRKY13* | Cotton_D_gene_10033299 | — | — |
| *GhWRKY120* | Gh_D02G0043 | III | *WRKY005* | Gorai.005G003900.1 | *WRKY005* | gene_10002578 | *GrWRKY31* | Cotton_D_gene_10002578 | *GhWRKY31* | KF669773 |
| *GhWRKY121* | Gh_D02G0605 | II-c | *WRKY006* | Gorai.005G068100.1 | *WRKY006* | gene_10005791 | *GrWRKY32* | Cotton_D_gene_10005791 | *GhWRKY32* | KF669809 |
| *GhWRKY122* | Gh_D02G1291 | II-c | *WRKY007* | *Gorai.005G144500.1* | *WRKY007* | gene_10037859 | *GrWRKY33* | Cotton_D_gene_10037859 | *GhWRKY33* | KF669810 |
| *GhWRKY123* | Gh_D02G1493 | II-c | *WRKY008* | Gorai.005G164300.1 | — | — | *GrWRKY34* | Cotton_D_gene_10024223 | *GhWRKY34* | KF669842 |
| *GhWRKY124* | Gh_D03G0226 | II-d | *WRKY009* | Gorai.003G026600.1 | — | — | *GrWRKY55* | Cotton_D_gene_10008614 | *GhWRKY55* | GU207869* |
| *GhWRKY125* | Gh_D03G0441 | II-c | *WRKY010* | Gorai.003G047800.1 | — | — | *GrWRKY20* | Cotton_D_gene_10036575 | *GhWRKY20* | KF669806 |
| *GhWRKY126* | Gh_D03G0444 | II-c | *WRKY011* | Gorai.003G048100.1 | *WRKY011* | gene_10036580 | *GrWRKY21* | Cotton_D_gene_10036580 | *GhWRKY21* | KF669807 |
| *GhWRKY127* | Gh_D03G0682 | II-e | *WRKY012* | Gorai.003G085500.1 | *WRKY012* | gene_10031091 | *GrWRKY19* | Cotton_D_gene_10031091 | *GhWRKY19* | KF669784 |
| *GhWRKY128* | Gh_D03G1371 | I | *WRKY013* | Gorai.003G150700.1 | *WRKY013* | gene_10012177 | *GrWRKY18* | Cotton_D_gene_10012177 | *GhWRKY18* | KF669858 |
| *GhWRKY129* | Gh_D04G0112 | III | *WRKY014* | Gorai.012G013900.1 | *WRKY014* | gene_10016711 | *GrWRKY103* | Cotton_D_gene_10016711 | *GhWRKY103* | KF669820 |
| *GhWRKY130* | Gh_D04G0113 | III | *WRKY015* | Gorai.012G014000.1 | *WRKY015* | gene_10016710 | *GrWRKY102* | Cotton_D_gene_10016710 | *GhWRKY102* | KF669772 |
| *GhWRKY131* | Gh_D04G0203 | I | *WRKY016* | Gorai.012G028200.1 | *WRKY016* | gene_10006792 | *GrWRKY109* | Cotton_D_gene_10006792 | — | — |
| *GhWRKY132* | Gh_D04G0323 | II-c | *WRKY017* | Gorai.012G040700.1 | *WRKY017* | gene_10019812 | *GrWRKY101* | Cotton_D_gene_10019812 | *GhWRKY101* | KF669777 |
| *GhWRKY133* | Gh_D04G0418 | I | *WRKY018* | Gorai.012G051500.1 | *WRKY018* | gene_10009963 | *GrWRKY3* | Cotton_D_gene_10009963 | *GhWRKY3* | KF669771 |
| *GhWRKY134* | Gh_D04G0470 | II-b | *WRKY019* | Gorai.012G056800.1 | *WRKY019* | gene_10027942 | *GrWRKY26* | Cotton_D_gene_10027942 | — | — |
| *GhWRKY135* | Gh_D04G0725 | II-c | *WRKY020* | Gorai.012G085100.1 | — | — | *GrWRKY100* | Cotton_D_gene_10001752 | *GhWRKY100* | KF669819 |
| *GhWRKY136* | Gh_D04G1122 | I | *WRKY036* | Gorai.009G421200.1 | *WRKY036* | gene_10027770 | *GrWRKY98* | Cotton_D_gene_10027770 | *GhWRKY98* | KF669818 |
| *GhWRKY137* | Gh_D04G1177 | II-c | *WRKY021* | Gorai.012G104800.1 | *WRKY021* | gene_10026013 | *GrWRKY99* | Cotton_D_gene_10026013 | *GhWRKY99* | KF669846 |
| *GhWRKY138* | Gh_D04G1318 | I | *WRKY022* | Gorai.012G119600.1 | *WRKY022* | gene_10016568 | *GrWRKY97* | Cotton_D_gene_10016568 | *GhWRKY97* | KF669852 |
| *GhWRKY139* | Gh_D04G1896 | I | *WRKY023* | Gorai.012G186000.1 | *WRKY023* | gene_10012245 | *GrWRKY40* | Cotton_D_gene_10012245 | *GhWRKY40* | KF669767 |
| *GhWRKY140* | Gh_D05G0600 | II-a | *WRKY024* | Gorai.009G062300.1 | *WRKY024* | gene_10037079 | *GrWRKY71* | Cotton_D_gene_10037079 | *GhWRKY71* | KF669857 |
| *GhWRKY141* | Gh_D05G0601 | II-a | *WRKY025* | Gorai.009G062400.1 | *WRKY025* | gene_10037078 | *GrWRKY70* | Cotton_D_gene_10037078 | *GhWRKY70* | KF669834 |
| *GhWRKY142* | Gh_D05G0648 | II-d | *WRKY026* | Gorai.009G066900.1 | *WRKY026* | gene_10037032 | *GrWRKY69* | Cotton_D_gene_10037032 | *GhWRKY69* | KF669845 |
| *GhWRKY143* | Gh_D05G1063 | II-e | *WRKY027* | Gorai.009G116800.1 | — | — | *GrWRKY72* | Cotton_D_gene_10003094 | *GhWRKY72* | KF669791 |
| *GhWRKY144* | Gh_D05G1137 | II-a | *WRKY028* | Gorai.009G124000.1 | *WRKY028* | gene_10007125 | *GrWRKY73* | Cotton_D_gene_10007125 | *GhWRKY73* | KF669835 |
| *GhWRKY145* | Gh_D05G1432 | II-c | *WRKY029* | Gorai.009G157300.1 | *WRKY029* | gene_10033628 | *GrWRKY74* | Cotton_D_gene_10033628 | *GhWRKY74* | KF669814 |
| *GhWRKY146* | Gh_D05G1645 | II-b | *WRKY031* | Gorai.009G180400.1 | *WRKY031* | gene_10033857 | *GrWRKY76* | Cotton_D_gene_10033857 | *GhWRKY76* | KF669827 |
| *GhWRKY147* | Gh_D05G1968 | II-d | *WRKY032* | Gorai.009G214800.1 | *WRKY032* | gene_10011482 | *GrWRKY94* | Cotton_D_gene_10011482 | *GhWRKY94* | KF669847 |
| *GhWRKY148* | Gh_D05G2344 | II-c | *WRKY033* | Gorai.009G259600.1 | *WRKY033* | gene_10023486 | *GrWRKY77* | Cotton_D_gene_10023486 | *GhWRKY77* | KF669816 |
| *GhWRKY149* | Gh_D05G2499 | II-d | *WRKY034* | Gorai.009G276100.1 | *WRKY034* | gene_10023655 | *GrWRKY78* | Cotton_D_gene_10023655 | *GhWRKY78* | KF669801 |
| *GhWRKY150* | Gh_D05G2642 | III | *WRKY035* | Gorai.009G292600.1 | *WRKY035* | gene_10016255 | *GrWRKY7* | Cotton_D_gene_10016255 | *GhWRKY7* | KF669776 |
| *GhWRKY151* | Gh_D05G3827 | II-e | *WRKY030* | Gorai.009G160800.1 | *WRKY030* | gene_10033661 | *GrWRKY75* | Cotton_D_gene_10033661 | — | — |
| *GhWRKY152* | Gh_D06G0174 | II-d | — | — | — | — | — | — | — | — |
| *GhWRKY153* | Gh_D06G0175 | II-d | *WRKY037* | Gorai.010G022600.1 | *WRKY037* | gene_10025883 | *GrWRKY81* | Cotton_D_gene_10025883 | *GhWRKY81* | KF669830 |
| *GhWRKY154* | Gh_D06G1029 | I | *WRKY039* | Gorai.010G113000.1 | *WRKY039* | gene_10039495 | *GrWRKY83* | Cotton_D_gene_10039495 | *GhWRKY83* | KF669836 |
| *GhWRKY155* | Gh_D06G1078 | II-a | *WRKY040* | Gorai.010G118300.1 | *WRKY040* | gene_10039553 | *GrWRKY84* | Cotton_D_gene_10039553 | *GhWRKY84* | KF669802 |
| *GhWRKY156* | Gh_D06G1082 | II-a | — | — | — | — | — | — | — | — |
| *GhWRKY157* | Gh_D06G1121 | II-d | *WRKY041* | Gorai.010G121800.1 | *WRKY041* | gene_10039598 | *GrWRKY85* | Cotton_D_gene_10039598 | *GhWRKY85* | KF669792 |
| *GhWRKY158* | Gh_D06G1196 | II-c | *WRKY042* | Gorai.010G131300.1 | *WRKY042* | gene_10015040 | *GrWRKY111* | Cotton_D_gene_10015040 | *GhWRKY111* | KF669800 |
| *GhWRKY159* | Gh_D06G1453 | II-e | *WRKY043* | Gorai.010G161000.1 | *WRKY043* | gene_10040769 | *GrWRKY86* | Cotton_D_gene_10040769 | *GhWRKY86* | KF669817 |
| *GhWRKY160* | Gh_D06G1486 | II-c | *WRKY044* | Gorai.010G164700.1 | *WRKY044* | gene_10040730 | *GrWRKY87* | Cotton_D_gene_10040730 | *GhWRKY87* | KF669829 |
| *GhWRKY161* | Gh_D06G1651 | II-b | *WRKY045* | Gorai.010G182500.1 | *WRKY045* | gene_10035091 | *GrWRKY88* | Cotton_D_gene_10035091 | *GhWRKY88* | KF669774 |
| *GhWRKY162* | Gh_D06G1939 | III | *WRKY046* | Gorai.010G219200.1 | *WRKY046* | gene_10005482 | *GrWRKY89* | Cotton_D_gene_10005482 | — | — |
| *GhWRKY163* | Gh_D06G1966 | II-a | *WRKY047* | Gorai.010G222400.1 | *WRKY047* | gene_10023185 | *GrWRKY1* | Cotton_D_gene_10023185 | *GhWRKY1* | KF669831 |
| *GhWRKY164* | Gh_D06G2328 | II-c | *WRKY038* | Gorai.010G098000.1 | *WRKY038* | gene_10026362 | *GrWRKY108* | Cotton_D_gene_10026362 | *GhWRKY108* | KF669765 |
| *GhWRKY165* | Gh_D07G0023 | II-d | *WRKY048* | Gorai.001G002200.1 | *WRKY048* | gene_10012784 | *GrWRKY12* | Cotton_D_gene_10012784 | *GhWRKY12* | KF669853 |
| *GhWRKY166* | Gh_D07G0177 | II-c | *WRKY049* | Gorai.001G021500.1 | *WRKY049* | gene_10015108 | *GrWRKY114* | Cotton_D_gene_10015108 | *GhWRKY114* | KF669805 |
| *GhWRKY167* | Gh_D07G0267 | I | *WRKY050* | Gorai.001G032200.1 | *WRKY050* | gene_10015280 | *GrWRKY10* | Cotton_D_gene_10015280 | *GhWRKY10* | KF669760 |
| *GhWRKY168* | Gh_D07G0317 | II-a | *WRKY051* | Gorai.001G037700.1 | *WRKY051* | gene_10015331 | *GrWRKY11* | Cotton_D_gene_10015331 | *GhWRKY11* | KF669832 |
| *GhWRKY169* | Gh_D07G0318 | II-a | *WRKY052* | Gorai.001G037800.1 | — | — | — | — | — | — |
| *GhWRKY170* | Gh_D07G0505 | II-c | *WRKY053* | Gorai.001G057600.1 | — | — | *GrWRKY9* | Cotton_D_gene_10022823 | *GhWRKY9* | KF669841 |
| *GhWRKY171* | Gh_D07G0824 | II-b | *WRKY054* | Gorai.001G094300.1 | *WRKY054* | gene_10023049 | *GrWRKY8* | Cotton_D_gene_10023049 | *GhWRKY8* | KF669823 |
| *GhWRKY172* | Gh_D07G1299 | II-b | *WRKY055* | Gorai.001G147900.1 | *WRKY055* | gene_10028569 | *GrWRKY6* | Cotton_D_gene_10028569 | *GhWRKY6* | KF669821 |
| *GhWRKY173* | Gh_D07G1384 | III | *WRKY056* | Gorai.001G155800.1 | *WRKY056* | gene_10028479 | *GrWRKY5* | Cotton_D_gene_10028479 | *GhWRKY5* | KF669781 |
| *GhWRKY174* | Gh_D07G1672 | I | *WRKY057* | Gorai.001G192900.1 | — | — | — | — | — | — |
| *GhWRKY175* | Gh_D07G1877 | II-b | *WRKY058* | Gorai.001G214800.1 | *WRKY058* | gene_10026164 | *GrWRKY4* | Cotton_D_gene_10026164 | *GhWRKY4* | KF669822 |
| *GhWRKY176* | Gh_D07G2107 | I | *WRKY059* | Gorai.001G241200.1 | *WRKY059* | gene_10001713 | *GrWRKY112* | Cotton_D_gene_10001713 | *GhWRKY112* | KF669803 |
| *GhWRKY177* | Gh_D07G2135 | II-d | *WRKY118* | Gorai.013G104500.1 | *WRKY118* | gene_10016391 | *GrWRKY116* | Cotton_D_gene_10016391 | *GhWRKY116* | KF669843 |
| *GhWRKY178* | Gh_D07G2328 | II-c | *WRKY060* | Gorai.001G273100.1 | *WRKY060* | gene_10019327 | *GrWRKY2* | Cotton_D_gene_10019327 | *GhWRKY2* | KF669759 |
| *GhWRKY179* | Gh_D08G0290 | I | *WRKY061* | Gorai.004G033200.1 | *WRKY061* | gene_10002745 | *GrWRKY22* | Cotton_D_gene_10002745 | *GhWRKY22* | KF669763 |
| *GhWRKY180* | Gh_D08G0306 | I | *WRKY062* | Gorai.004G034600.1 | *WRKY062* | gene_10002760 | *GrWRKY24* | Cotton_D_gene_10002760 | *GhWRKY24* | KF669764 |
| *GhWRKY181* | Gh_D08G0620 | II-c | *WRKY063* | Gorai.004G069500.1 | — | — | *GrWRKY45* | Cotton_D_gene_10020122 | *GhWRKY45* | KF669840 |
| *GhWRKY182* | Gh_D08G1232 | III | *WRKY064* | Gorai.004G134600.1 | *WRKY064* | gene_10007498 | *GrWRKY27* | Cotton_D_gene_10007498 | *GhWRKY27* | KF669775 |
| *GhWRKY183* | Gh_D08G1333 | II-c | *WRKY065* | Gorai.004G144900.1 | *WRKY065* | gene_10022405 | *GrWRKY104* | Cotton_D_gene_10022405 | — | — |
| *GhWRKY184* | Gh_D08G1424 | II-c | *WRKY066* | Gorai.004G155100.1 | *WRKY066* | gene_10025638 | *GrWRKY62* | Cotton_D_gene_10025638 | — | — |
| *GhWRKY185* | Gh_D08G1474 | II-e | *WRKY067* | Gorai.004G160100.1 | — | — | — | — | — | — |
| *GhWRKY186* | Gh_D08G1543 | II-c | *WRKY068* | Gorai.004G166800.1 | *WRKY068* | gene_10029195 | *GrWRKY25* | Cotton_D_gene_10029195 | *GhWRKY25* | KF669808 |
| *GhWRKY187* | Gh_D08G1841 | II-d | *WRKY069* | Gorai.004G199500.1 | *WRKY069* | gene_10009592 | *GrWRKY28* | Cotton_D_gene_10009592 | *GhWRKY28* | KF669796 |
| *GhWRKY188* | Gh_D08G2032 | II-d | *WRKY070* | Gorai.004G219300.1 | — | — | *GrWRKY29* | Cotton_D_gene_10005114 | *GhWRKY29* | KF669795 |
| *GhWRKY189* | Gh_D08G2279 | II-d | *WRKY072* | Gorai.004G247000.1 | *WRKY072* | gene_10007968 | *GrWRKY23* | Cotton_D_gene_10007968 | *GhWRKY23* | KF669794 |
| *GhWRKY190* | Gh_D09G0067 | II-c | *WRKY073* | Gorai.006G008200.1 | *WRKY073* | gene_10027029 | *GrWRKY37* | Cotton_D_gene_10027029 | *GhWRKY37* | KF669811 |
| *GhWRKY191* | Gh_D09G0113 | I | *WRKY074* | Gorai.006G013300.1 | *WRKY074* | gene_10027087 | *GrWRKY36* | Cotton_D_gene_10027087 | *GhWRKY36* | FJ966887* |
| *GhWRKY192* | Gh_D09G0213 | II-b | *WRKY075* | Gorai.006G025700.1 | *WRKY075* | gene_10014412 | *GrWRKY35* | Cotton_D_gene_10014412 | — | — |
| *GhWRKY193* | Gh_D09G0376 | II-c | *WRKY076* | Gorai.006G043200.1 | — | — | — | — | — | — |
| *GhWRKY194* | Gh_D09G1456 | II-c | *WRKY077* | Gorai.006G171700.1 | *WRKY077* | gene_10017279 | *GrWRKY38* | Cotton_D_gene_10017279 | *GhWRKY38* | KF669838 |
| *GhWRKY195* | Gh_D09G2332 | II-c | *WRKY078* | Gorai.006G265200.1 | *WRKY078* | gene_10021341 | *GrWRKY39* | Cotton_D_gene_10021341 | *GhWRKY39* | KF669812 |
| *GhWRKY196* | Gh_D10G0061 | II-e | *WRKY079* | Gorai.011G006600.1 | *WRKY079* | gene_10031158 | *GrWRKY90* | Cotton_D_gene_10031158 | *GhWRKY90* | KF669851 |
| *GhWRKY197* | Gh_D10G0117 | II-b | *WRKY080* | Gorai.011G012700.1 | *WRKY080* | gene_10031219 | *GrWRKY91* | Cotton_D_gene_10031219 | *GhWRKY91* | KF669793 |
| *GhWRKY198* | Gh_D10G0329 | II-e | *WRKY081* | Gorai.011G037500.1 | *WRKY081* | gene_10031462 | *GrWRKY92* | Cotton_D_gene_10031462 | *GhWRKY92* | KF669849 |
| *GhWRKY199* | Gh_D10G0367 | II-c | *WRKY082* | Gorai.011G041400.1 | *WRKY082* | gene_10031500 | *GrWRKY93* | Cotton_D_gene_10031500 | *GhWRKY93* | KF669854 |
| *GhWRKY200* | Gh_D10G0757 | II-c | *WRKY083* | Gorai.011G086300.1 | — | — | *GrWRKY95* | Cotton_D_gene_10010798 | *GhWRKY95* | KF669855 |
| *GhWRKY201* | Gh_D10G1011 | II-c | *WRKY084* | Gorai.011G114200.1 | *WRKY084* | gene_10030213 | *GrWRKY110* | Cotton_D_gene_10030213 | — | — |
| *GhWRKY202* | Gh_D10G1797 | II-c | *WRKY085* | Gorai.011G201800.1 | *WRKY085* | gene_10015718 | *GrWRKY96* | Cotton_D_gene_10015718 | *GhWRKY96* | KF669769 |
| *GhWRKY203* | Gh_D11G0118 | II-e | *WRKY086* | Gorai.007G013300.1 | *WRKY086* | gene_10017815 | *GrWRKY57* | Cotton_D_gene_10017815 | *GhWRKY57* | KF669787 |
| *GhWRKY204* | Gh_D11G0132 | III | *WRKY087* | Gorai.007G014600.1 | *WRKY087* | gene_10017802 | *GrWRKY56* | Cotton_D_gene_10017802 | *GhWRKY56* | KF669779 |
| *GhWRKY205* | Gh_D11G0976 | II-e | *WRKY088* | Gorai.007G103400.1 | *WRKY088* | gene_10035636 | *GrWRKY48* | Cotton_D_gene_10035636 | *GhWRKY48* | KF669785 |
| *GhWRKY206* | Gh_D11G0977 | II-c | *WRKY089* | Gorai.007G103600.1 | *WRKY089* | gene_10035639 | *GrWRKY49* | Cotton_D_gene_10035639 | *GhWRKY49* | KF669813 |
| *GhWRKY207* | Gh_D11G1011 | III | *WRKY090* | Gorai.007G107300.1 | *WRKY090* | gene_10035678 | *GrWRKY50* | Cotton_D_gene_10035678 | *GhWRKY50* | KF669783 |
| *GhWRKY208* | Gh_D11G1099 | II-b | *WRKY091* | Gorai.007G117200.1 | *WRKY091* | gene_10035779 | *GrWRKY51* | Cotton_D_gene_10035779 | *GhWRKY51* | KF669825 |
| *GhWRKY209* | Gh_D11G1141 | II-d | *WRKY093* | Gorai.007G122300.1 | *WRKY093* | gene_10035830 | *GrWRKY53* | Cotton_D_gene_10035830 | *GhWRKY53* | KF669786 |
| *GhWRKY210* | Gh_D11G1328 | II-d | *WRKY094* | Gorai.007G142800.1 | *WRKY094* | gene_10009679 | *GrWRKY47* | Cotton_D_gene_10009679 | *GhWRKY47* | KF669798 |
| *GhWRKY211* | Gh_D11G1536 | I | *WRKY095* | Gorai.007G167100.1 | *WRKY095* | gene_10014637 | *GrWRKY46* | Cotton_D_gene_10014637 | *GhWRKY46* | KF669766 |
| *GhWRKY212* | Gh_D11G1584 | II-b | *WRKY096* | Gorai.007G171900.1 | *WRKY096* | gene_10004689 | *GrWRKY43* | Cotton_D_gene_10004689 | — | — |
| *GhWRKY213* | Gh_D11G1963 | II-d | *WRKY097* | Gorai.007G216700.1 | *WRKY097* | gene_10036922 | *GrWRKY42* | Cotton_D_gene_10036922 | *GhWRKY42* | KF669797 |
| *GhWRKY214* | Gh_D11G2246 | II-c | *WRKY099* | Gorai.007G246500.1 | — | — | *GrWRKY115* | Cotton_D_gene_10015628 | *GhWRKY115* | KF669839 |
| *GhWRKY215* | Gh_D11G2566 | II-d | *WRKY100* | Gorai.007G278400.1 | *WRKY100* | gene_10008065 | *GrWRKY54* | Cotton_D_gene_10008065 | *GhWRKY54* | KF669799 |
| *GhWRKY216* | Gh_D12G0371 | II-b | *WRKY101* | Gorai.008G041400.1 | *WRKY101* | gene_10009488 | *GrWRKY82* | Cotton_D_gene_10009488 | *GhWRKY82* | KF669768 |
| *GhWRKY217* | Gh_D12G0971 | II-c | *WRKY102* | Gorai.008G109600.1 | — | — | *GrWRKY66* | Cotton_D_gene_10014946 | *GhWRKY66* | KF669848 |
| *GhWRKY218* | Gh_D12G1207 | II-b | *WRKY103* | Gorai.008G134300.1 | *WRKY103* | gene_10035431 | *GrWRKY79* | Cotton_D_gene_10035431 | *GhWRKY79* | KF669828 |
| *GhWRKY219* | Gh_D12G1243 | II-c | *WRKY104* | Gorai.008G137900.1 | *WRKY104* | gene_10035475 | *GrWRKY80* | Cotton_D_gene_10035475 | *GhWRKY80* | KF669815 |
| *GhWRKY220* | Gh_D12G1253 | II-d | — | — | — | — | — | — | — | — |
| *GhWRKY221* | Gh_D12G1614 | II-c | *WRKY106* | Gorai.008G178100.1 | *WRKY106* | gene_10014899 | *GrWRKY67* | Cotton_D_gene_10014899 | *GhWRKY67* | KF669844 |
| *GhWRKY222* | Gh_D12G1734 | II-b | *WRKY107* | Gorai.008G191200.1 | *WRKY107* | gene_10006708 | *GrWRKY68* | Cotton_D_gene_10006708 | *GhWRKY68* | KF669826 |
| *GhWRKY223* | Gh_D12G1827 | II-c | *WRKY108* | Gorai.008G200800.1 | *WRKY108* | gene_10029943 | *GrWRKY63* | Cotton_D_gene_10029943 | — | — |
| *GhWRKY224* | Gh_D12G2301 | III | *WRKY111* | Gorai.008G253300.1 | *WRKY111* | gene_10016858 | *GrWRKY60* | Cotton_D_gene_10016858 | *GhWRKY60* | KF669778 |
| *GhWRKY225* | Gh_D12G2429 | II-e | — | — | — | — | — | — | — | — |
| *GhWRKY226* | Gh_D12G2748 | II-e | *WRKY110* | Gorai.008G250300.1 | *WRKY110* | gene_10016888 | *GrWRKY61* | Cotton_D_gene_10016888 | *GhWRKY61* | KF669790 |
| *GhWRKY227* | Gh_D13G0071 | III | *WRKY114* | Gorai.013G008300.1 | *WRKY114* | gene_10024748 | *GrWRKY107* | Cotton_D_gene_10024748 | — | — |
| *GhWRKY228* | Gh_D13G0744 | II-b | *WRKY115* | Gorai.013G082300.1 | *WRKY115* | gene_10033488 | *GrWRKY105* | Cotton_D_gene_10033488 | *GhWRKY105* | KF669770 |
| *GhWRKY229* | Gh_D13G0820 | I | *WRKY116* | Gorai.013G090200.1 | *WRKY116* | gene_10033582 | *GrWRKY106* | Cotton_D_gene_10033582 | *GhWRKY106* | KF669780 |
| *GhWRKY230* | Gh_D13G0837 | II-c | *WRKY117* | Gorai.013G092300.1 | *WRKY117* | gene_10019857 | *GrWRKY41* | Cotton_D_gene_10019857 | — | — |
| *GhWRKY231* | Gh_D13G1418 | I | *WRKY119* | Gorai.013G155500.1 | *WRKY119* | gene_10006909 | *GrWRKY14* | Cotton_D_gene_10006909 | *GhWRKY14* | KF669762 |
| *GhWRKY232* | Gh_D13G1636 | I | *WRKY120* | Gorai.013G179400.1 | *WRKY120* | gene_10000655 | *GrWRKY113* | Cotton_D_gene_10000655 | *GhWRKY113* | KF669804 |
| *GhWRKY233* | Gh_Sca005564G01 | II-c | *WRKY092* | Gorai.007G121200.1 | *WRKY092* | gene_10035819 | *GrWRKY52* | Cotton_D_gene_10035819 | *GhWRKY52* | KF669850 |
| *GhWRKY234* | Gh_Sca005611G01 | II-d | — | — | — | — | — | — | — | — |
| *GhWRKY235* | Gh_Sca005611G02 | II-d | *WRKY071* | Gorai.004G219400.1 | *WRKY071* | gene_10005113 | *GrWRKY30* | Cotton_D_gene_10005113 | *GhWRKY30* | KF669856 |
| *GhWRKY236* | Gh_Sca005978G01 | I | — | — | — | — | — | — | — | — |
| *GhWRKY237* | Gh_Sca009206G01 | II-e | *WRKY113* | Gorai.008G273700.1 | *WRKY113* | gene_10030785 | *GrWRKY58* | Cotton_D_gene_10030785 | *GhWRKY58* | KF669788 |
| *GhWRKY238* | Gh_Sca010421G01 | Ⅳ | — | — | — | — | — | — | — | — |
| *GhWRKY239* | Gh_Sca204211G01 | Ⅳ | — | — | — | — | — | — | — | — |
