# Supplementary material for: Identification of the group IIa WRKY subfamily and the functional analysis of GhWRKY17 in upland cotton (Gossypium hirsutum L.)
Source: PLoS One. 2018 Jan 25;13(1):e0191681. doi: 10.1371/journal.pone.0191681 (PMC5784973; doi:10.1371/journal.pone.0191681)
Supplement: S3 Table — (DOCX) [file pone.0191681.s007.docx]

| **Group** | **Number** |
| --- | --- |
| Group I | 36 |
| Group II-a | 15 |
| Group II-b | 32 |
| Group II-c | 75 |
| Group II-d | 34 |
| Group II-e | 23 |
| Group III | 21 |
| Group Ⅳ | 3 |
| Total | 239 |
